# Demographic and epidemiological characteristics of pregnant and postpartum women who died from severe acute respiratory syndrome in Brazil: A retrospective cohort study comparing COVID-19 and nonspecific etiologic causes

**DOI:** 10.1371/journal.pone.0274797

**Published:** 2022-10-03

**Authors:** Veridiana Freire Franco, Agatha Sacramento Rodrigues, Elias Ribeiro Rosa Junior, Luciana Graziela de Godói, Nátaly Adriana Jimenez Monroy, Rafaela Alkmin da Costa, Rossana Pulcineli Vieira Francisco

**Affiliations:** 1 Departamento de Obstetrícia e Ginecologia of Hospital das Clínicas HCFMUSP, Faculdade de Medicina, Universidade de São Paulo, São Paulo, Brazil; 2 Departamento de Estatística da Universidade Federal do Espírito Santo, Vitória–Espírito Santo, Brazil; Federal University of Sao Carlos: Universidade Federal de Sao Carlos, BRAZIL

## Abstract

The objective of this study is to compare the demographic characteristics and symptoms in pregnant and postpartum women who died from Severe Acute Respiratory Syndrome (SARS) caused by COVID-19 or by nonspecific cause in different states of Brazil. This is a retrospective cohort study and the analysis was conducted on SARS death records between 02/16/2020 and 04/17/2021, obtained from the Information System for the Epidemiological Surveillance of Influenza (Sistema de Informação da Vigilância Epidemiológica da Gripe, SIVEP-Gripe). Pregnant and postpartum women, aged between 10 and 55 years, who died from SARS, were included and classified into two groups: SARS due to confirmed COVID-19 or SARS due to nonspecific cause. The cases were analyzed according to the women’s demographic and epidemiological characteristics, clinical symptoms, risk factors and disease evolution. As results, 19,333 pregnant and postpartum women were identified. From these, 1,279 died (1,026 deaths from COVID-19 and 253 deaths from SARS with nonspecific cause). The groups showed significant differences in age, education, race, and occurrence of obesity and chronic lung disease. The group of women who died from confirmed COVID-19 presented a significantly higher frequency of symptoms of fever, cough, fatigue, loss of taste, and loss of smell, as well as a higher rate of admission to the intensive care unit (ICU). Data analysis draws attention to the high number of cases of SARS without a causal diagnosis, the low access to ICU and orotracheal intubation (OTI), which might be explained by the demographic and regional inequalities in the access to healthcare.

## Introduction

COVID-19 (Coronavirus disease 2019), as named by the World Health Organization (WHO), is a syndrome caused by a new coronavirus (SARS-CoV-2), whose outbreak began in the city of Wuhan, China, at the end of December 2019 [1]. On March 11, 2020, COVID-19 was characterized by the WHO as a pandemic. As of June 24, 2021, 179,065,823 cases of SARS-CoV-2 infection and 3,886,347 deaths caused by this coronavirus have been confirmed worldwide [2]. SARS-CoV-2 belongs to the Coronavirus (CoV) family. This is a family of RNA viruses, known since the 1960s, that affects the human respiratory tract. SARS-CoV-2 can be present asymptomatically or cause a broad spectrum of respiratory or gastrointestinal symptoms that can lead to severe and potentially fatal conditions. Transmission occurs through direct or indirect contact with infected individuals, through saliva and respiratory secretions [3].

At first, pregnant and postpartum women were not considered at higher risk, since most of the initial cases were classified as mild or moderate and only a small portion required intensive care or mechanical ventilation. However, the epidemiological bulletin released by the Brazilian Ministry of Health in May 2020 already showed 252 cases of Severe Acute Respiratory Syndrome (SARS) in pregnant women, with 36 maternal deaths confirmed by COVID-19 [4].

In Brazil, up to the second half of June 2021, 18,054,653 COVID-19 cases (and more than 500,000 deaths) have been confirmed [2]; however, underreporting of cases is possible, either due to unavailability of the diagnostic test, the lost of the window of opportunity for testing or even the quality of the registry of laboratorial data. Until April 2020, the estimated notification rate (number of confirmed cases reported by the national Ministry of Health divided by the number of cases estimated from the number of deaths) in Brazil was 9.2%, suggesting that less than 10% of COVID-19 cases were being diagnosed and that the notification of confirmed cases in Brazil is far below those of other countries such as the United States, Spain, or Italy, whose notification rate is above 15%, or South Korea and Germany where more than 50% of predicted cases had a proper diagnosis [5]. With this high underreporting rate (90.8% of cases might be unreported), several deaths caused by SARS-CoV-2 may have been unreported or may have been identified as nonspecific SARS, generating also underreporting of maternal death from SARS-CoV-2. Remarkably, according to data from the Information System for the Epidemiological Surveillance of Influenza (Sistema de Informação da Vigilância Epidemiológica da Gripe, SIVEP-Gripe), a Brazilian nationwide healthcare surveillance database, the number of deaths related to SARS in pregnant/postpartum women with nonspecific cause was 50 in 2019 and this number increased nearly 5-fold in 2020/2021 [6].

In this study, we analyzed the records of death cases of pregnant and postpartum women due to SARS, caused either by COVID-19 or by nonspecific cause, and compared the sociodemographic characteristics and clinical presentation of both groups of patients. We also analyzed the ratio of maternal death by SARS caused by COVID-19 or by nonspecific cause over the different states of Brazil.

## Methods

This study consisted of a retrospective cohort analysis of data obtained through the Information System for the Epidemiological Surveillance of Influenza (Sistema de Informação da Vigilância Epidemiológica da Gripe, SIVEP-Gripe) database. SIVEP-Gripe collects the records of hospitalization and death from SARS. Notification is compulsory for flu-like illness, which is characterized by at least two signs and symptoms: fever, even if reported, chills, sore throat, headache, cough, runny nose, and olfactory or taste disorders, plus dyspnea / respiratory discomfort, persistent pressure in the chest, oxygen (O_2_) saturation lower than 95% in room air, or bluish color of lips or face. Records of deaths of asymptomatic individuals with laboratory confirmation by molecular biology or immunological examination for COVID-19 infection were also collected.

SIVEP-Gripe should be notified of all hospitalization cases in Brazilian public or private hospitals and all deaths due to severe acute respiratory infections, regardless of hospitalization. SARS surveillance has been carried out by the Brazilian Ministry of Health through the Health Surveillance Secretariat (Secretaria de Vigilância em Saúde, SVS) since the Influenza A (H1N1) pandemic in 2009.

We selected the cases notified between 2020 and 2021 that met the definition of SARS by SIVEP-Gripe. The first records of COVID-19 in Brazil date from the 8^th^ epidemiological week of 2020. For this reason, the analyzed period comprises epidemiological data from weeks 8 to 53 of 2020 (02/16/2020–01/02/2021) and weeks 1 to 15 of 2021 (01/03/2021–04/17/2021). The database was obtained from the official website of the Ministry of Health of Brazil on 05/05/2021 [6]. The study included data from pregnant and postpartum women aged between 10 and 55 years who evolved to death due to SARS, either by confirmed COVID-19 or by nonspecific cause. The unspecified SARS definition in SIVEP- GRIPE database is" a SARS case for which no other etiological agent was identified OR it was not possible to collect/process a clinical sample for laboratory diagnosis, OR that it was not possible to confirm by clinical epidemiological, clinical-imaging or clinical criteria. Those subjects with SARS etiology field left in blank were not included in the analysis because we could not determine if they met the unspecified SARS definition or if it was a registry quality issue. Cases of SARS confirmed as Influenza, other virus or other etiologic agent were also excluded.

Chosen variables were: age, gestational moment (first trimester, second trimester, third trimester, or puerperium), type and number of comorbidities (heart disease, kidney disease, neurological disease, hematologic disease, liver disease, diabetes, asthma, obesity, chronic lung disease, and immunosuppression), educational level, self-reported skin color (white, black, brown, yellow, or indigenous), area of residence (peri-urban, rural, or urban), symptoms (fever, cough, sore throat, dyspnea, respiratory discomfort, O_2_ saturation <95%, at least one respiratory symptom, diarrhea, vomiting, abdominal pain, fatigue, loss of smell or taste), nosocomial case, intensive care unit (ICU) admission, and ventilatory support.

Since SIVEP-Gripe is an open database, the information we used are from public domain and with no possibility of individual identification, according to Brazilian regulations of the National Research Ethics Comission (Comissão Nacional de Ética em Pesquisa–CONEP), this study does not require prior approval by the institutional ethics board (data in S1 Text) [7].

To better visualize the spatial distribution of deaths resulting from cases classified as COVID-19 or nonspecific, maps of death ratios (number of maternal deaths during a given time period per 100,000 live births during the same time) were constructed for each of the Brazilian states. To calculate the number of live births in each state, the 2019 SINASC database (Sistema de Informações sobre Nascidos Vivos) was used [8]. This database records all births in Brazilian territory.

All analyzes and maps were conducted using the free statistical software R (R Foundation for Statistical Computing Platform, version 4.0.3). Maps were produced using the shapefiles from geobr package(data in S1 Dataset) [9].

### Data analysis

We analyzed the SIVEP-Gripe notifications of SARS that evolved to death, due to confirmed COVID-19 or due to nonspecific cause. A chi-square test was performed to determine the association between the analyzed variables and the classification of cases as COVID-19 or nonspecific. When the observed or expected frequency was less than 5 cases, a Fisher’s exact test was performed instead. Additionally, the Odds Ratios (OR) and respective confidence intervals (95% CI) were calculated. The medians of the quantitative variables were compared using the nonparametric Wilcoxon-Mann-Whitney test. In cases where the quantitative variables showed an approximate symmetric behavior, the comparison of means was performed using a t-test. All inferential analyzes were considered significant at a 5% threshold.

To better visualize the spatial distribution of deaths resulting from cases classified as COVID-19 or nonspecific, maps of death rates (number of maternal deaths during a given time period per 100,000 live births during the same time)were constructed or each of the Brazilian states. To calculate the number of live births in each state, the 2019 SINASC database (Sistema de Informações sobre Nascidos Vivos) was used [8]. This database records all births in Brazilian territory.

All analyzes and maps were conducted using the free statistical software R (R Foundation for Statistical Computing Platform, version 4.0.3).

## Results

**Fig 1:**
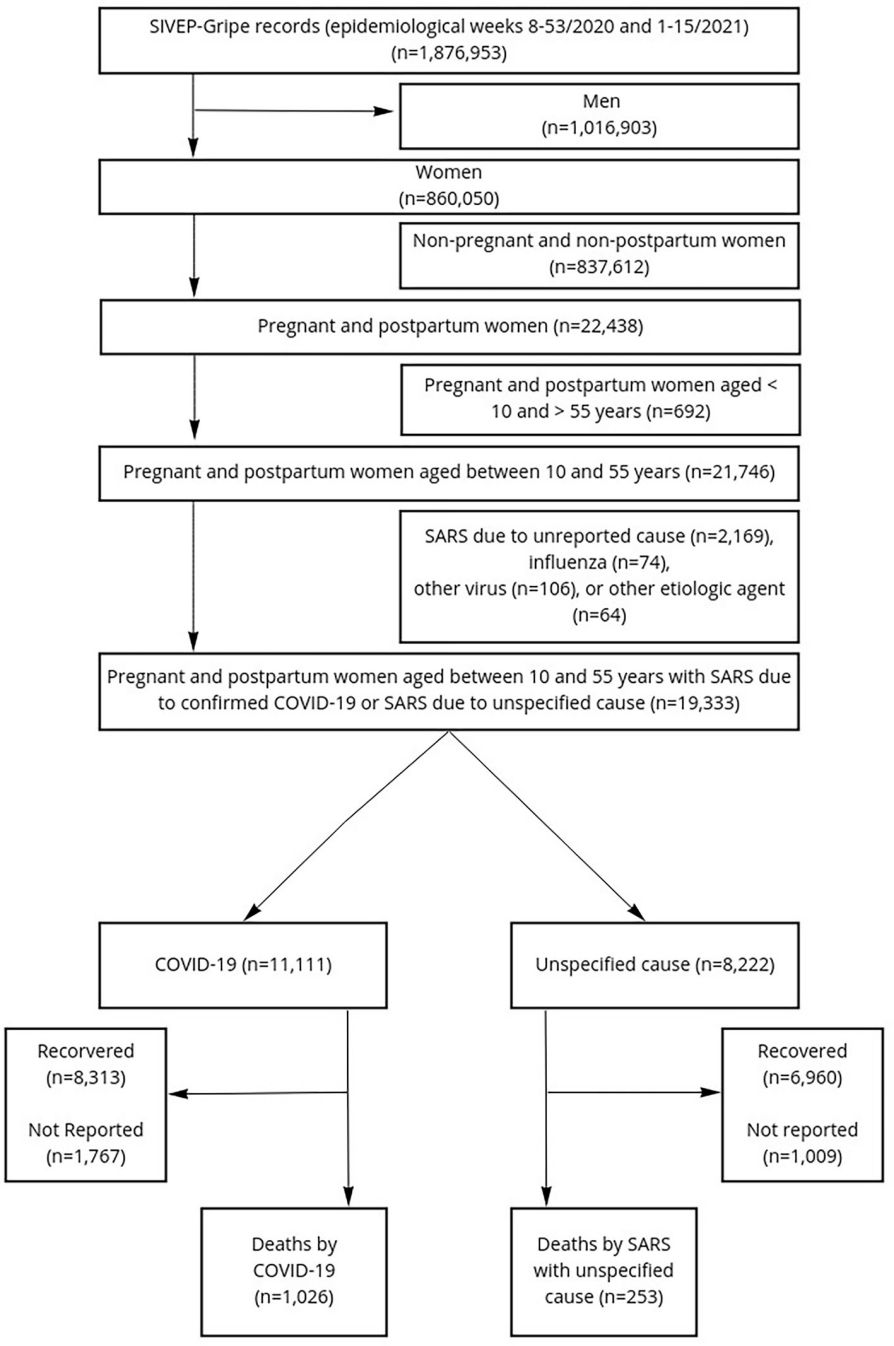
Case selection flowchart.

We identified 19,333 cases of pregnant and postpartum women aged between 10 and 55 years with SARS diagnosis, either due to confirmed COVID-19 or of nonspecific cause. From these, 1,279 women died. These cases were classified into two groups according to the cause of death: deaths from COVID-19 (n = 1,026) and deaths from SARS of nonspecific cause (n = 253) (Fig 1).

**Fig 2:**
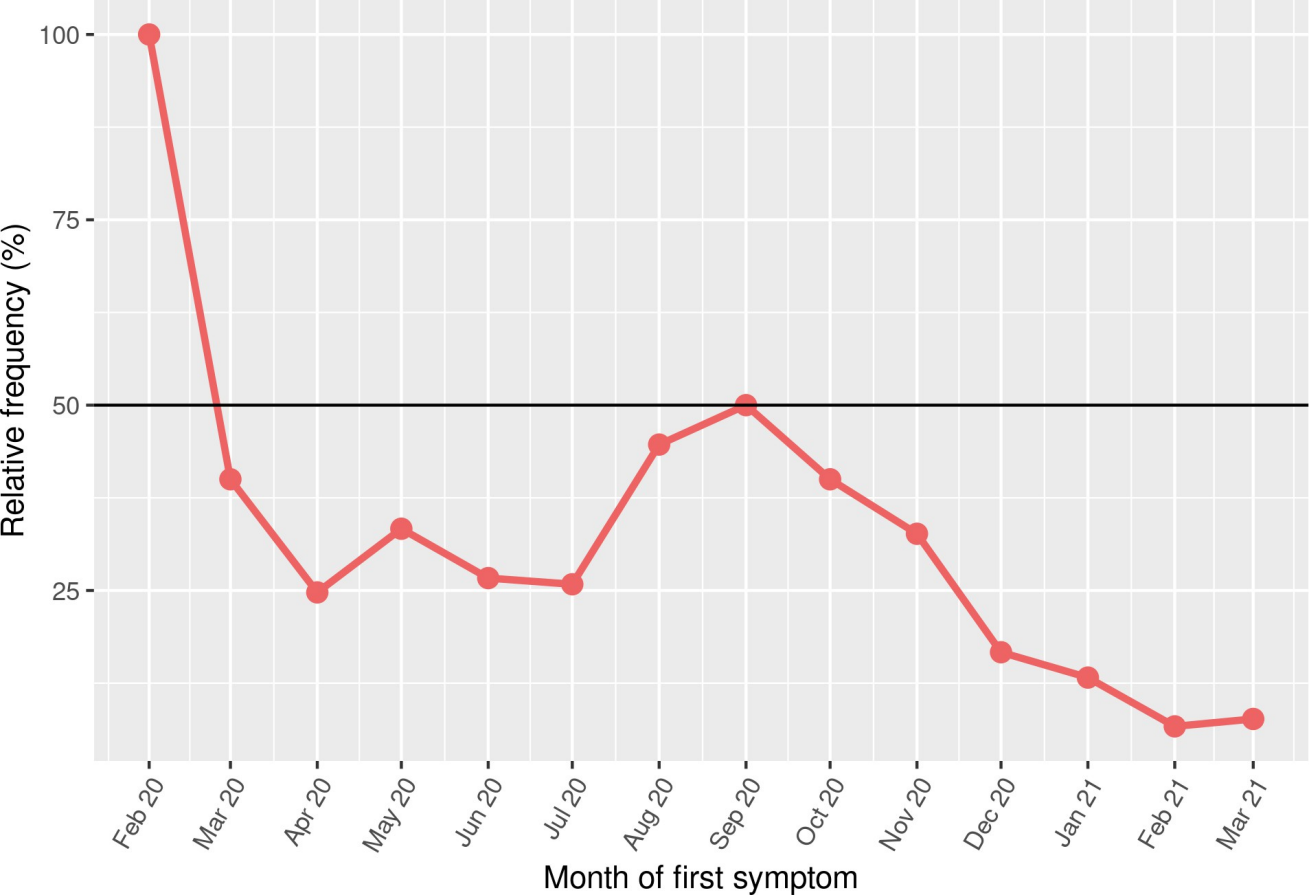
Percentage of cases of SARS with unspecified cause according to the date of the first symptom of COVID-19, reported from February 2020 to March 2021 (elaborated by the authors). Percentages are relative to total cases of SARS in pregnant or postpartum women, which means that percentage of SARS cases due to COVID-19 is the complement of the proportion represented by each point.

From September 2020 onwards, there was a constant decrease in the proportion of deaths classified as nonspecific (Fig 2). Since we have only two classification groups, the complement of the proportion represented by each point corresponds to the deaths classified as due to COVID-19. In the first analyzed month (February 2020), all deaths (n = 3) corresponded to nonspecific cases of SARS; this proportion dropped to approximately 10% in the last month analyzed (March 2021), meaning that the remaining 90% of deaths were COVID-19-related cases of SARS.

These groups showed significant differences in age, education, and race (Table 1). The mean maternal age was higher in the group of patients with death confirmed by COVID-19 than in the group with death from nonspecific cause (32.24 ± 7.49 years vs. 30.19 ± 9.09 years; p = 0.001). The proportion of pregnant and postpartum women with higher education studies was higher in the group with death confirmed by COVID-19 and the proportion of women with no education or primary education was higher in the nonspecific cause group. Regarding race, we noticed a higher proportion of women of black race in the group with death from nonspecific cause than in the COVID-19 group, while the other races were present with similar percentages in both groups (Table 1).

**Table 1:** Demographic characteristics of pregnant and postpartum women who died from SARS according to the cause (COVID-19 or nonspecific).

Characteristics of patients	SARS caused by COVID-19	Nonspecific SARS	p-value for comparison
**Age (years)**			
Mean ± S.D.	32.24 ± 7.49	30.19 ± 9.09	**0.0010** ^**a**^
**Age group (years)**	**N (%)**	**N (%)**	**<0.0001** ^**b**^
<20 (N = 67)	36 (3.5%)	31 (12.3%)	
20–34 (N = 737)	593 (57.8%)	144 (56.9%)	
≥35 (N = 475)	397 (38.7%)	78 (30.8%)	
**Race/Color**	**N (%)**	**N (%)**	**0.0183** ^**b**^
White (N = 384)	311 (34.3%)	73 (34.3%)	
Black (N = 105)	73 (8.0%)	32 (15.0%)	
Yellow (N = 12)	11 (1.2%)	1 (0.5%)	
Brown (N = 609)	505 (55.7%)	104 (48.8%)	
Indigenous (N = 10)	7 (0.8%)	3 (1.4%)	
**Education**	**N (%)**	**N (%)**	**<0.0001** ^**b**^
No education (N = 8)	4 (0.8%)	4 (4.3%)	
Primary Education (N = 157)	121 (25.,1%)	36 (38.,3%)	
Secondary Education (N = 311)	262 (54.4%)	49 (52.1%)	
Higher Education (N = 100)	95 (19.7%)	5 (5.3%)	
**Residence area**	**N (%)**	**N (%)**	0.0398 ^c^
Rural (N = 104)	75 (8.2%)	29 (12.6%)	
Urban (N = 1150)	845 (91.8%)	201 (87.4%)	
**Gestational moment**	**N (%)**	**N (%)**	**<0.0001** ^**b**^
1^st^ trimester (N = 72)	45 (4.4%)	27 (10.7%)	
2^nd^ trimester (N = 259)	214 (20.9%)	45 (17.8%)	
3^rd^ trimester (N = 432)	376 (36.6%)	56 (22.1%)	
Unknown (N = 50)	42 (4.1%)	8 (3.2%)	
Puerperium (N = 466)	349 (34.0%)	117 (46.2%)	

Primary education corresponds to nine school years. Secondary education corresponds to 12 school years and Higher education corresponds to >12 school years.

^a^ Student t-test

^b^ Fisher’s exact test

^c^ Chi-square test

In the analyzed groups, the occurrence of obesity was significantly different (24.2% vs. 10.9%; p = 0.0017), being more prevalent in SARS deaths caused by COVID-19. The occurrence of chronic lung disease between the groups was also significantly different (2.0% vs. 7.0%; p = 0.0071): the chance of having a case of chronic lung disease among the deaths of SARS caused by COVID-19 was approximately one quarter than among deaths of nonspecific causes of SARS (OR 0.27; 95% CI: 0.11–0.67) (Table 2).

**Table 2:** Frequency of comorbidities in pregnant and postpartum women who died from SARS according to the cause (COVID-19 or nonspecific).

Comorbidity	SARS caused by COVID-19 (N(%))	Nonspecific SARS (N(%))	p-value	OR (95% CI)
Heart disease (N = 670)	118/529 (22.3%)	43/141 (30.5%)	0.0559^a^	0.65 (0.43–1.00)
Diabetes (N = 670)	117/534 (21.9%)	24/136 (17.6%)	0.3315^a^	1.31 (0.81–2.13)
Obesity (N = 662)	129/534 (24.2%)	14/128 (10.9%)	**0.0017** ^a^	**2.59 (1.44–4.68)**
Asthma (N = 643)	43/513 (8.4%)	8 /130 (6.2%)	0.5101^a^	1.40 (0.64–3.05)
Chronic hematological disease (N = 636)	13/508 (2.6%)	6/128 (4.7%)	0.3302^a^	0.53 (0.20–1.43)
Chronic liver disease (N = 631)	7/502 (1.4%)	3/129 (2.3%)	0.4349^b^	0.59 (0.13–3.61)
Chronic kidney disease (N = 627)	13/498 (2.6%)	5/129 (3.9%)	0.3904^b^	0.67 (0.22–2.43)
Neurological disease (N = 630)	6/502 (1.2%)	5/128 (3.9%)	0.5196^b^	0.30 (0.074–1.26)
Chronic lung disease (N = 631)	10/503 (2.0%)	9/128 (7.0%)	**0.0071** ^a^	**0.27 (0.11–0.67)**
Immunodepression (N = 635)	22/506 (4.3%)	7/129 (5.4%)	0.7737^a^	0.79 (0.33–1.90)

^a^ Chi-square test/

^b^Fisher’s exact test

When comparing the type of symptoms in both groups, we observed more reported symptoms in patients that died from SARS caused by COVID-19, namely fever (68.9% vs. 55.9%; p<0.0001), cough (79.4% vs. 57.8%; p <0.0001), fatigue (35.7% vs. 19.4%; p = 0.0023), loss of smell (16.4% vs. 5.2%; p = 0.0028), and loss of taste (15.2% vs. 5.2%; p = 0.0062) (Table 3). In particular, the prevalence of loss of smell and taste among the cases of confirmed COVID-19 is more than three times higher than in cases of nonspecific cause. The presence of hospital-acquired infection was also significantly different (3.2% vs. 7.1%; p = 0.0264), being higher in the group of deaths from nonspecific cause. Symptoms such as dyspnea, respiratory distress, O_2_ saturation, and others did not show significant differences between groups.

**Table 3:** Frequency of symptoms in pregnant and postpartum women who died from SARS according to the cause (COVID-19 or nonspecific).

Symptom	SARS caused by COVID-19 (N(%))	Nonspecific SARS (N(%))	p value	OR (95% CI)
Fever (N = 1091)	611/887 (68.9%)	114/204 (55.9%)	**< 0.0001** ^**a**^	**1.75 (1.28–2.38)**
Cough (N = 1129)	729/918 (79.4%)	122/211 (57.8%)	**< 0.0001** ^**a**^	**2.81 (2.05–3.86)**
Throat pain (N = 929)	193/750 (25.7%)	33/179 (18.4%)	0.0515^**a**^	1.53 (1.02–2.31)
Dyspnea (N = 1164)	807/939 (85.9%)	191/225 (84.9%)	0.7643^**a**^	1.09 (0.72–1.64)
Respiratory discomfort (N = 1112)	690/893 (77.3%)	173/219 (79.0%)	0.6461^**a**^	0.90 (0.63–1.30)
O_2_ saturation <95% (N = 1101)	695/890 (78.1%)	153/211 (72.5%)	0.1009^**a**^	1.35 (0.96–1.90)
Diarrhea (N = 919)	110/741 (14.8%)	20/178 (11.2%)	0.2624^**a**^	1.38 (0.83–2.29)
Vomiting (N = 916)	91/733 (12.4%)	33/183 (18.0%)	0.062^**a**^	0.64 (0.42–1.00)
Abdominal pain (N = 652)	59/554 (10.6%)	10/98 (10.2%)	0.9999^**a**^	1.05 (0.52–2.13)
Fatigue (N = 670)	204/572 (35.7%)	19/98 (19.4%)	**0.0023** ^**a**^	**2.30 (1.36–3.91)**
Loss of smell (N = 658)	92/562 (16.4%)	5/96 (5.2%)	**0.0028** ^**b**^	**3.56 (1.41–11.52)**
Loss of taste (N = 660)	86/564 (15.2%)	5/96 (5.2%)	**0.0062** ^**b**^	**3.27 (1.30–10.61)**

^a^ Chi-square test/

^b^Fisher’s exact test

Among the pregnant or postpartum women who died from SARS, those of the COVID-19 group presented a higher rate of admission to the ICU (77.6% vs. 69.2%; p = 0.0104). The frequency of orotracheal intubation (OTI) was similar between groups (66.6% vs. 60.0%; p = 0.0746).

Among patients admitted to ICU, both the mean duration of hospitalization and the time elapsed between the start of symptoms and the date of death were significantly longer for the COVID-19 group (13.61 ± 14.25 days vs. 7.70 ± 12.88 days, p<0.001, and 20.00 ± 15.04 days vs. 11.53 ± 13.45 days, p<0.001, respectively).

**Fig 3:**
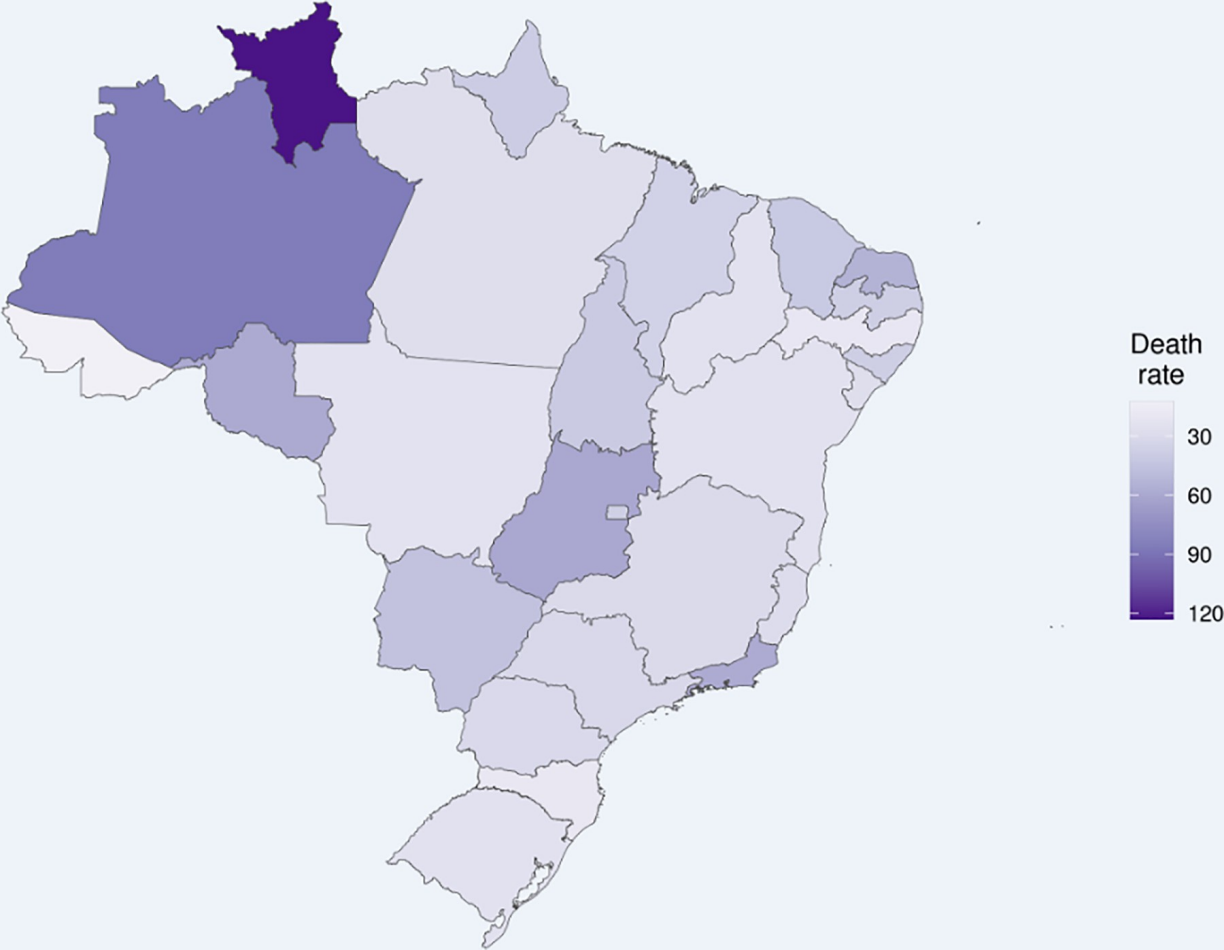
Maternal death ratio by COVID-19 in Brazilian states (elaborated by the authors). Maternal death ratio was calculated using the number of maternal deaths/100,000 live births, which was estimated from the number of live births from 2019 from SINASC database. Each state is colored according to tones of purple according to the shown scale. The number of live births in each state in 2019 was considered for this estimate (http://svs.aids.gov.br/dantps/centrais-de-conteudos/paineis-de-monitoramento/natalidade/nascidos-vivos/).

**Fig 4:**
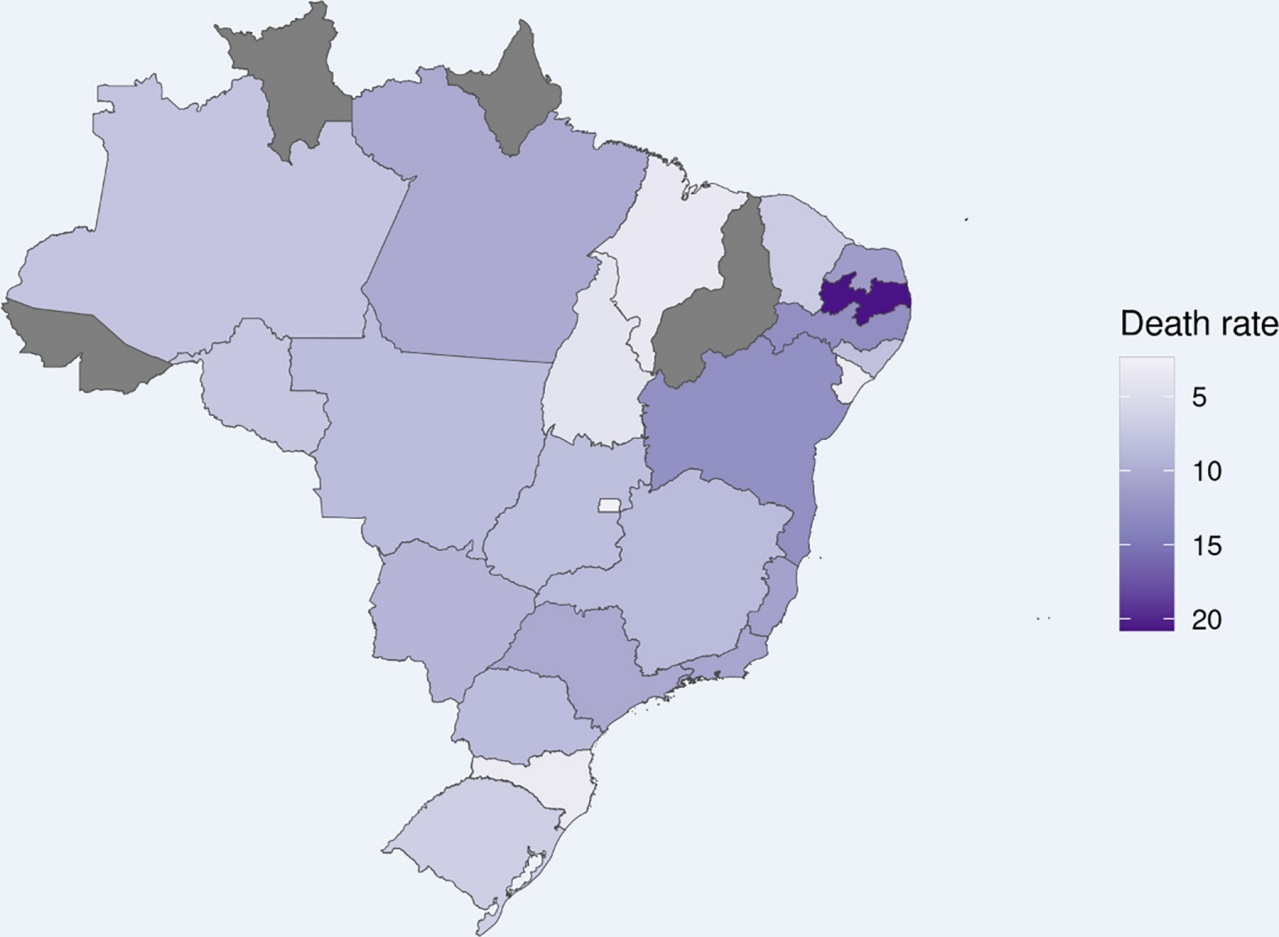
Maternal death ratio by unspecified cause in Brazilian states (elaborated by the authors). Maternal death ratio was calculated using the number of maternal deaths/100,000 live births, which was estimated from the number of live births from 2019 from SINASC database. Each state is colored according to tones of purple according to the shown scale. The number of live births in each state in 2019 was considered for this estimate (http://svs.aids.gov.br/dantps/centrais-de-conteudos/paineis-de-monitoramento/natalidade/nascidos-vivos/).

The maternal death ratio (number of maternal deaths/100,000 live births–estimated from the number of live births from 2019) by COVID-19 (Fig 3) and by unidentified cause (Fig 4) is heterogeneous throughout Brazil. The ratios of maternal death due to COVID-19 are higher in the states of Roraima and Amazonas (north), and the ratio of death due to nonspecific causes is higher in Paraíba and Pernambuco and Bahia (northeast).

## Discussion

### Principal findings

From February 2020 to April 2021, 1,279 maternal deaths from SARS were registered, 253 (19.8%) of which had no specific cause. There was a reduction in the number of cases of nonspecific cause during the evaluated period. In addition, uneven distribution of the nonspecific cases over the different Brazilian states was observed (Fig 3), which may point to lower testing or greater difficulties in accessing healthcare services in certain areas. It highlights sociodemographic and clinical aspects related to limited healthcare access, from diagnosis until receiving treatment, that pregnant women in Brazil face.

At the beginning of the pandemic, the lack of sufficient testing and the consequent overcrowding of health services may have influenced the rates of death from nonspecific cause. With the advance of the pandemic and the greater testing availability, there was a decrease in the number of deaths from nonspecific SARS, but even in the most recent months analyzed (February and March 2021) we still observed 7.7% of such cases (Fig 2). This decrease can be attributed to more testing being done in pregnant women after they were classified as a risk group.

Some demographic and clinical factors were associated with nonspecific SARS cases. For example, the frequency of lower educational levels was higher among pregnant and postpartum women with deaths from nonspecific cause. The frequency of women under 20 years of age was higher in the unspecific cause of death group than in the COVID-19 group, as was the percentage of black women (Table 1). Previous studies have associated higher fertility rates and not having a health insurance plan with disadvantaged sociodemographic and economic characteristics in Brazil, such as lower maternal age, not being white, being unemployed, having less schooling and residing in the North region of the country [10]. These disadvantages are characteristics of the inequity associated with less access to the health system [11].

Deaths from COVID-19 were higher among black Brazilian women [12]. However, special attention should be paid to the greater frequency of black women in the nonspecific cause group of SARS. Black women tend to have less schooling and more restricted access to health services [13–15]. It suggests less appreciation of symptoms and even less access to diagnostic tests in this population than white women and women. Together, our findings of higher frequencies of younger, black and with lower education level women in the nonspecific cause group leads us to infer that this group comprises women with poor access to the health system and that may justify their lack of etiological diagnosis of SARS.

Besides, nonwhite ethnicity, unemployment, lower education level, lower income, living in the North region, rural living status, and having no health insurance are characteristics of inequity associated with smaller access to healthcare system [11]. Together, our findings of higher frequencies of younger, black and with lower education level women in the nonspecific cause group leads us to infer that this group comprises women with poor access to the health system and that may justify their lack of etiological diagnosis of SARS.

Additionally, obesity was 2.59 times more frequent in the group of deaths from COVID-19 (Table 2). One explanation is that this condition was one of the first to be identified as a risk factor for worse evolution of COVID-19, which may have led to the prioritization of the obese population in the COVID-19 diagnosis [12, 16]. Also, the higher frequency of lung disease in the group of death from nonspecific cause of SARS may have occurred because some SARS symptoms could have been misunderstood as a clinical exacerbation of the baseline disease and, therefore, COVID-19 diagnostic test could have been more neglected in such cases.

Cases with typical symptoms were most frequently associated with SARS-CoV-2 infection and they could have been used as a criterion for diagnostic testing. Symptoms such as loss of smell and taste are hallmarks of coronavirus infection. Although they are not pathognomonic, their wide dissemination in the media increased the demand for testing. Besides being a symptom of COVID-19, fever is also considered a sign for awareness or a severity marker when occurring in pregnant women. This fact may have increased the search for a number of diagnostic tests in cases of fever, which also happened in cases of coughing, described as the second most common symptom after fever in COVID-19 [17].

In the studied population, confirmed COVID-19 patients presented more typical symptoms of SARS than those with nonspecific cause. The frequency of admission to the ICU, the need for ventilation support, and the average number of days in the intensive care unit were all higher in patients who died from COVID-19-caused SARS, and that could reflect a better quality of healthcare received by this group of women, indicated by a higher access to diagnosis tests and, possibly, to more and better therapeutic resources and, consequently, longer hospital stay. On the other hand, access to the ICU (77.6% vs. 69.2%) and the OTI (66.6% vs. 60.0%) were extremely low in both groups, considering that only fatal cases were selected for analysis.

We also observed that, although the deaths from nonspecific causes decreased, these remained very high in certain states and contributed to increasing maternal death ratios in Brazilian states. This distribution reflects the already known inequalities in access to healthcare and the lack of infrastructures in hospitals and lack of ICU beds, which was an important reason for the high number of maternal deaths in Brazil. The same factors may have caused an uneven distribution of SARS deaths both from COVID-19 and from nonspecific cause (Fig 3). Studies with specific and standardized methodological procedures addressing COVID-19 infection in the obstetric population may help to understand the gaps highlighted in here.

### Limitations of the study

This study evaluated a high number of cases of maternal death, using a national database of hospitalized patients with data collection since the beginning of the COVID-19 pandemic in Brazil. As a nationwide database study, it has some limitations, mainly related to the level of completeness and quality of information added to the SIVEP-gripe form fields, with numerous cases of missing data. Besides, as a population study, it does not allow us to infer the causality among variables, nor to address the quality of healthcare provided to each group, but it can raise the hypothesis that the group without access to a proper etiologic diagnosis also presents some unfavorable socioeconomic features and could also had a poorer access to SARS treatment.

It is important to emphasize that, over time, greater knowledge of the disease and the emergence of more test options allowed for an increase in the number of diagnoses. However, we still have low testing of patients in Brazil and a large number of cases classified as unspecified.

## Conclusions

The data presented here draws attention to the high number of cases of SARS-related deaths without a final etiological diagnosis in Brazil, specially in subjects with sociodemographic features frequently associated with limited access to healthcare, such as black ethnicity, younger age and low educational level. Besides, restrictions to proper access to healthcare is toughened by the even lower admission rate in ICU among women that died for unspecified-SARS.

## Supporting information

S1 Text Brazilian regulations of the National Research Ethics Comission. (DOCX)

S1 Dataset Codes and ouputs of article. (PDF)
