# Supplementary material for: Demographic and epidemiological characteristics of pregnant and postpartum women who died from severe acute respiratory syndrome in Brazil: A retrospective cohort study comparing COVID-19 and nonspecific etiologic causes
Source: PLoS One. 2022 Oct 3;17(10):e0274797. doi: 10.1371/journal.pone.0274797 (PMC9529093; doi:10.1371/journal.pone.0274797)
Supplement: S1 Text — (DOCX) [file pone.0274797.s001.docx]

**Research ethics committee approval**

Since SIVEP-Gripe is an open database, with no possibility of individual identification, according to Brazilian regulations of the National Research Ethics Comission (Comissão Nacional de Ética em Pesquisa – CONEP), this study does not require prior approval by the institutional ethics board.

The Resolution is transcripted below:

“Esta Resolução dispõe sobre as normas aplicáveis a pesquisas em Ciências
Humanas e Sociais cujos procedimentos metodológicos envolvam a utilização de dados diretamente obtidos com os participantes ou de informações identificáveis ou que possam acarretar riscos maiores do que os existentes na vida cotidiana, na forma definida nesta Resolução.

Parágrafo único. Não serão registradas nem avaliadas pelo sistema CEP/CONEP:

(...)

V - pesquisa com bancos de dados, cujas informações são agregadas, sem
possibilidade de identificação individual.”

Translated to English:

"This Resolution provides the standards applicable to research in Human and Social Sciences which methodological procedures involve the use of data directly obtained from the participants or from identifiable information or which may entail greater risks than those existing in everyday life, in the form defined in this Resolution.

Single paragraph. Will not be registered or evaluated by the Ethical Committee:

(…)

V - search with databases which information is aggregated without possibility of individual identification.”

Reference: Brasil. Ministério da Saúde. Conselho Nacional de Saúde. Resolução nº 510, de 7 de abril de 2016. Diário Oficial da União. Brasília, 24/05/2016. Available in: <https://www.in.gov.br/materia/-/asset_publisher/Kujrw0TZC2Mb/content/id/22917581>. [Accessed in Sep 09th, 2021]
