# Supplementary material for: Demographic and epidemiological characteristics of pregnant and postpartum women who died from severe acute respiratory syndrome in Brazil: A retrospective cohort study comparing COVID-19 and nonspecific etiologic causes
Source: PLoS One. 2022 Oct 3;17(10):e0274797. doi: 10.1371/journal.pone.0274797 (PMC9529093; doi:10.1371/journal.pone.0274797)
Supplement: S1 Dataset — (PDF) [file pone.0274797.s002.pdf]

Demographic and epidemiological characteristics of pregnant and postpartum women who died from Severe Acute Respiratory Syndrome in Brazil: a comparison between COVID-19 and nonspecific etiologic causes

Codes and outputs

02/10/2022

Contents

Description **2**

R packages used, functions and dataset import **2**

Case selection and data treatment **4**

Epidemiologic characteristics **7**

Comorbities **11**

Symptoms **20**

Outcome **31**

Distribution of COVID-19 and unspecified etiologic cause by Brazilian states **34**

Distribution by Epidemiological Week **37**

Description

This file presents the documentation of the analysis of article "Demographic and epidemiological characteristics of pregnant and postpartum women who died from Severe Acute Respiratory Syndrome in Brazil: a comparison between COVID-19 and nonspecific etiologic causes".

R packages used, functions and dataset import

The data are analyzed using the free-software R (https://www.R-project.org) in version 4.0.3. Next, we present and load the libraries used in the data analysis process.

```
#load packages
loadlibrary <- function(x) {
  if (!require(x, character.only = TRUE)) {
    install.packages(x, dependencies = T)
    if (!require(x, character.only = TRUE))
      stop("Package not found")
  }
}

packages <-
  c(
    "dplyr",
    "lubridate",
    "readr",
    "readxl",
    "ggplot2",
    "kableExtra",
    "tables",
    "questionr",
    "car",
    "data.table",
    "magrittr",
    "tidyverse",
    "readxl",
    "summarytools",
    "modelsummary",
    "RColorBrewer",
    "zoo",
    "grid",
    "gridExtra",
    "cowplot",
    "effectsize",
    "rcompanion",
    "DescTools",
    "geobr",
    "scales"
  )
lapply(packages, loadlibrary)
```

One can see below the functions that will be used in the data analysis.

```r
#functions for summary measures
media <- function(x)
  mean(x, na.rm = TRUE)
mediana <- function(x)
  median(x, na.rm = TRUE)
DP <- function(x)
  sd(x, na.rm = TRUE)
minimo <- function(x)
  base::min(x, na.rm = TRUE)
maximo <- function(x)
  base::max(x, na.rm = TRUE)
q25 <- function(x)
  stats::quantile(x, p = 0.25, na.rm = TRUE)
q75 <- function(x)
  stats::quantile(x, p = 0.75, na.rm = TRUE)
IQR <- function(x)
  round(q75(x) - q25(x), 2)
n <- function(x)
  sum(!is.na(x))
```

The Influenza Epidemiological Surveillance Information System, SIVEP-Gripe (Sistema de Informação de Vigilância Epidemiológica da Gripe), is a nationwide surveillance database used to monitor severe acute respiratory infections in Brazil.

Notification is mandatory for Influenza Syndrome (characterized by at least two of the following signs and symptoms: fever, even if referred, chills, sore throat, headache, cough, runny nose, olfactory or taste disorders) and who has dyspnea/respiratory discomfort or persistent pressure in the chest or O2 saturation less than 95% in room air or bluish color of the lips or face. Asymptomatic individuals with laboratory confirmation by molecular biology or immunological examination for COVID-19 infection are also reported.

For notifications in Sivep-Gripe, hospitalized cases in both public and private hospitals and all deaths due to severe acute respiratory infections regardless of hospitalization must be considered.

The search was limited to the first notified case of COVID-19 in February 2020 until the 15th epidemiological week of 2021 (up to April 17, 2021). The datasets were obtained on May 5, 2021, on the site https://opendatasus.saude.gov.br/dataset. The first period (8th to 53rd epidemiological week of 2020) and the second period (1st to 15th epidemiological week of 2021) datasets can be obtained at https://drive.google.com/file/d/1jts4h0ovdwFh86SdKyslMLSG9rOy3UjX/view?usp=sharing and at https://drive.google.com/file/d/1gQSy_dcUkd1UrDEcsrDbyGl4gEvcI8z_/view?usp=sharing, respectively. The data are loaded below:

```r
#loading the datasets
#2021
data_2021 <- read_delim(
  "INFLUD21-03-05-2021.csv",
  ";",
  escape_double = FALSE,
  locale = locale(encoding = "ISO-8859-2"),
  trim_ws = TRUE
)

#2020
data_2020 <- read_delim(
  "INFLUD-03-05-2021.csv",
  ";",
  escape_double = FALSE,
```

```
  locale = locale(encoding = "ISO-8859-2"),
  trim_ws = TRUE
)

sem <- 15 #limit of epidemiological week of 2021

#### Concatenating 2020 and 2021 data ##############
data_all <- rbind(data_2020, data_2021)

# Creating the case year variable
data_all <-  data_all %>%
  dplyr::mutate(
    dt_sint = as.Date(DT_SIN_PRI, format = "%d/%m/%Y"),
    year_case = lubridate::year(dt_sint)
  )
```

There are 1905854 cases in the complete dataset. The case selection is presented in the following according to the flowchart presented in the article.

Case selection and data treatment

The first filter consists of selecting the cases from 8th epidemiological week of 2020 to 15th epidemiological week of 2021.

```
#Cases from the 8th epidemiological week of 2020
data1 <- data_all %>%
  filter((year_case == 2020 & SEM_PRI >= 8) | year_case == 2021)
```

There are 12563 cases in 2021 in epidemiological week 53 of 2020. These are cases from the first two days of 2021, which are still part of the last epidemiological week of 2020 (http://portalsinan.saude.gov.br/calendario-epidemiologico?layout=edit&id=168). However, these cases belong to the 53rd week of 2020 and we corrected as follows:

```
data2 <- data1 %>%
  mutate(year_case = ifelse(year_case == 2021 & SEM_PRI ==53, 2020, year_case)) %>%
  filter(year_case == 2020 | (year_case == 2021 & SEM_PRI <= sem))
```

There are 1876953 observations.

The next selection consists of selecting females cases:

```
#filtering F cases
data3 <- filter(data2, CS_SEXO == "F")
```

There are 860050 observations.

The next step is to identify pregnant and postpartum people (variable `classi_gesta_puerp`) and then select only those cases.

```r
#Creating the classification variable if pregnant, postpartum and not (neither pregnant nor postpartum)
data3 <- data3 %>%
  mutate(
    classi_gesta_puerp = case_when(
      CS_GESTANT == 1  ~ "1tri", #1st trimester
      CS_GESTANT == 2  ~ "2tri", #2st trimester
      CS_GESTANT == 3  ~ "3tri", #3st trimester
      CS_GESTANT == 4  ~ "GA_ig", #ignored gestational Age
      CS_GESTANT == 5 &
        PUERPERA == 1 ~ "puerp", #puerperium
      CS_GESTANT == 9 & PUERPERA == 1 ~ "puerp",  #puerperium
      TRUE ~ "no" #neither pregnant nor postpartum
    )
  )

#Filtering only pregnant and postpartum women
data4 <- data3 %>%
  filter(classi_gesta_puerp != "no")
```

There are 22438 observations.

The next selection consists of selecting women between 10 and 55 years old.

```r
# Filtering ages between 10 and 55 years old
data5 <- data4 %>%
  filter(TP_IDADE == 3 | (TP_IDADE != 3 & NU_IDADE_N >=8) #NU_IDADE_N is the age variable
         ) %>%
  filter(NU_IDADE_N > 9 & NU_IDADE_N <= 55)
```

There are 21746 observations.

Now we are going to select only the cases confirmed by COVID-19 (CLASSI_FIN = 5) or unspecified (CLASSI_FIN = 4). The other cases are influenza(CLASSI_FIN = 1), other virus(CLASSI_FIN = 2) and other etiologic agent(CLASSI_FIN = 3).

```r
freq(data5$CLASSI_FIN)
```

```
## Frequencies
## data5$CLASSI_FIN
## Type: Numeric
##
##                  Freq   % Valid   % Valid Cum.   % Total   % Total Cum.
## ----------- -------- --------- -------------- --------- --------------
##           1       74      0.38           0.38      0.34           0.34
##           2      106      0.54           0.92      0.49           0.83
##           3       64      0.33           1.25      0.29           1.12
##           4     8222     42.00          43.24     37.81          38.93
##           5    11111     56.76         100.00     51.09          90.03
##        <NA>     2169                               9.97         100.00
##       Total    21746    100.00         100.00    100.00         100.00
```

Now we are going to select only the cases confirmed by COVID-19 (CLASSI_FIN = 5) or unspecified (CLASSI_FIN = 4).

```r
#Filtering only covid or unspecified cases
data6 <- data5 %>%
    filter(CLASSI_FIN == 5 | CLASSI_FIN ==4)
```

Now labeling group variable:

```r
data6 <- data6 %>%
  mutate(group = case_when(
    CLASSI_FIN ==5 ~ "covid-19",
    TRUE ~ "unspecified"
  ))
data6$group <- factor(data6$group,
                      levels = c("unspecified","covid-19"))
```

There are 19333 observations.

We are now going to select only the death cases. The variable is `EVOLUCAO`, with 1 - cure, 2 - death by SARS, 3 - death by other cause.

```r
data6 <- data6 %>%
  mutate(death = case_when(
    EVOLUCAO == 1 ~ "cure",
    EVOLUCAO == 2 ~ "death",
    EVOLUCAO == 3 ~ "death",
    TRUE ~ NA_character_
  ))

with(data6, ctable(death, group,  prop = "c", chisq = FALSE))
```

```
## Cross-Tabulation, Column Proportions
## death * group
## Data Frame: data6
##
## ------- ------- --------------- ---------------- ----------------
##           group     unspecified         covid-19            Total
##   death
##    cure            6960 ( 84.7%)    8318 ( 74.9%)   15278 ( 79.0%)
##   death             253 (  3.1%)    1026 (  9.2%)    1279 (  6.6%)
##    <NA>            1009 ( 12.3%)    1767 ( 15.9%)    2776 ( 14.4%)
##   Total            8222 (100.0%)   11111 (100.0%)   19333 (100.0%)
## ------- ------- --------------- ---------------- ----------------
```

```r
data7 <- data6 %>%
  filter((EVOLUCAO == 2 | EVOLUCAO == 3) & !is.na(EVOLUCAO))
```

There are 1279 observations.

```r
with(data7, freq(group))
```

```
## Frequencies
## data7$group
```

```
## Type: Factor
##
##                      Freq   % Valid   % Valid Cum.   % Total   % Total Cum.
## ----------------- ------ --------- -------------- --------- --------------
##       unspecified    253     19.78          19.78     19.78          19.78
##          covid-19   1026     80.22         100.00     80.22         100.00
##              <NA>      0                               0.00         100.00
##             Total   1279    100.00         100.00    100.00         100.00
```

Epidemiologic characteristics

```
# Ethnicity
data7 <-  data7 %>%
  mutate(
    ethnicity = case_when(
      CS_RACA == 1 ~ "white",
      CS_RACA == 2 ~ "black",
      CS_RACA == 3 ~ "yellow",
      CS_RACA == 4 ~ "brown",
      CS_RACA == 5 ~ "indigenous",
      TRUE ~ NA_character_
    )
  )

# Education
data7 <-  data7 %>%
  mutate(education = case_when(CS_ESCOL_N == 0 ~ "no education",
                             CS_ESCOL_N == 1 | CS_ESCOL_N == 2 ~ "primary education",
                             CS_ESCOL_N == 3 ~ "secundary education",
                             CS_ESCOL_N == 4 ~ "higher education",
                               TRUE ~ NA_character_))

data7$education <- factor(data7$education,
                     levels = c("no education","primary education",
                                "secundary education", "higher education"))

# Age group
data7 <-  data7 %>%
  mutate(
    age_group = case_when(
      NU_IDADE_N <= 19 ~ "<20",
      NU_IDADE_N >= 20
      & NU_IDADE_N <= 34 ~ "20-34",
      NU_IDADE_N >= 35 ~ ">=35",
      TRUE ~ NA_character_
    )
  )
data7$age_group <-
  factor(data7$age_group, levels = c("<20", "20-34", ">=35"))
```

```r
# Residence area
data7 <-  data7 %>%
  mutate(zone = case_when(CS_ZONA ==1 | CS_ZONA == 3 ~ "urban",
                           CS_ZONA == 2 ~ "rural",
                             TRUE ~ NA_character_))
```

Ethnicity

```r
with(data7, ctable(ethnicity, group,  prop = "c", useNA = "no", chisq = FALSE))
```

```
## Cross-Tabulation, Column Proportions
## ethnicity * group
## Data Frame: data7
##
## ------------ ------- -------------- -------------- ---------------
##                group    unspecified       covid-19           Total
##    ethnicity
##        black             32 ( 15.0%)    73 (  8.0%)    105 (  9.4%)
##        brown            104 ( 48.8%)   505 ( 55.7%)    609 ( 54.4%)
##   indigenous              3 (  1.4%)     7 (  0.8%)     10 (  0.9%)
##        white             73 ( 34.3%)   311 ( 34.3%)    384 ( 34.3%)
##       yellow              1 (  0.5%)    11 (  1.2%)     12 (  1.1%)
##        Total            213 (100.0%)   907 (100.0%)   1120 (100.0%)
## ------------ ------- -------------- -------------- ---------------
```

```r
with(data7, fisher.test(ethnicity, group))
```

```
##
##  Fisher's Exact Test for Count Data
##
## data:  ethnicity and group
## p-value = 0.01825
## alternative hypothesis: two.sided
```

Education

```r
with(data7, ctable(education, group, prop = "c", useNA = "no", chisq = FALSE))
```

```
## Cross-Tabulation, Column Proportions
## education * group
## Data Frame: data7
##
## --------------------- ------- ------------- -------------- --------------
##                         group   unspecified       covid-19          Total
##              education
##           no education            4 (  4.3%)     4 (  0.8%)     8 (  1.4%)
##      primary education           36 ( 38.3%)   121 ( 25.1%)   157 ( 27.3%)
##    secundary education           49 ( 52.1%)   262 ( 54.4%)   311 ( 54.0%)
```

```
##      higher education            5 (  5.3%)    95 ( 19.7%)   100 ( 17.4%)
##                 Total           94 (100.0%)   482 (100.0%)   576 (100.0%)
## --------------------- -------- ------------- -------------- --------------
```

```
with(data7, fisher.test(education, group))
```

```
##
##  Fisher's Exact Test for Count Data
##
## data:  education and group
## p-value = 5.825e-05
## alternative hypothesis: two.sided
```

Age

```
datasummary((group) ~  NU_IDADE_N*(n+media+DP+mediana+q25+q75+IQR),
            data = data7, output = 'markdown')
```

	n	media	DP	mediana	q25	q75	IQR
unspecified	253.00	30.19	9.09	30.00	23.00	36.00	13.00
covid-19	1026.00	32.24	7.49	32.00	27.00	37.00	10.00

```
#t test
t.test(NU_IDADE_N ~ group, data = data7)
```

```
##
##  Welch Two Sample t-test
##
## data:  NU_IDADE_N by group
## t = -3.3239, df = 341.18, p-value = 0.0009841
## alternative hypothesis: true difference in means between group unspecified and group covid-19 is not
## 95 percent confidence interval:
##  -3.2662744 -0.8377097
## sample estimates:
## mean in group unspecified    mean in group covid-19
##                  30.18972                  32.24172
```

```
#effect size
c_cohen <- cohens_d(NU_IDADE_N ~ as.factor(group),data=data7)
c_cohen
```

```
## Cohen's d |         95% CI
## --------------------------
## -0.26     | [-0.40, -0.12]
##
## - Estimated using pooled SD.
```

```r
interpret_d(c_cohen$Cohens_d,rules="cohen1988")
```

```
## [1] "small"
## (Rules: cohen1988)
```

Age group

```r
with(data7, ctable(age_group, group, prop = "c", useNA = "no", chisq = FALSE))
```

```
## Cross-Tabulation, Column Proportions
## age_group * group
## Data Frame: data7
##
## ----------- ------- -------------- --------------- ---------------
##               group    unspecified        covid-19           Total
##   age_group
##         <20             31 ( 12.3%)     36 (  3.5%)     67 (  5.2%)
##       20-34            144 ( 56.9%)    593 ( 57.8%)    737 ( 57.6%)
##        >=35             78 ( 30.8%)    397 ( 38.7%)    475 ( 37.1%)
##       Total            253 (100.0%)   1026 (100.0%)   1279 (100.0%)
## ----------- ------- -------------- --------------- ---------------
```

```r
with(data7, fisher.test(age_group, group))
```

```
##
##  Fisher's Exact Test for Count Data
##
## data:  age_group and group
## p-value = 8.568e-07
## alternative hypothesis: two.sided
```

Residence area

```r
with(data7, ctable(zone, group, prop = "c", useNA = "no", chisq = FALSE))
```

```
## Cross-Tabulation, Column Proportions
## zone * group
## Data Frame: data7
##
## ------- ------- -------------- -------------- ---------------
##           group    unspecified       covid-19           Total
##    zone
##   rural             29 ( 12.6%)    75 (  8.2%)    104 (  9.0%)
##   urban            201 ( 87.4%)   845 ( 91.8%)   1046 ( 91.0%)
##   Total            230 (100.0%)   920 (100.0%)   1150 (100.0%)
## ------- ------- -------------- -------------- ---------------
```

```r
with(data7, fisher.test(zone, group))
```

```
##
##  Fisher's Exact Test for Count Data
##
## data:  zone and group
## p-value = 0.03982
## alternative hypothesis: true odds ratio is not equal to 1
## 95 percent confidence interval:
##  0.9921969 2.6039278
## sample estimates:
## odds ratio
##   1.624768
```

Gestational moment

```r
with(data7, ctable(classi_gesta_puerp, group, prop = "c", useNA = "no", chisq = TRUE))
```

```
## Cross-Tabulation, Column Proportions
## classi_gesta_puerp * group
## Data Frame: data7
##
##
## -------------------- ------- -------------- --------------- ---------------
##                        group    unspecified        covid-19           Total
##   classi_gesta_puerp
##                 1tri              27 ( 10.7%)     45 (  4.4%)     72 (  5.6%)
##                 2tri              45 ( 17.8%)    214 ( 20.9%)    259 ( 20.3%)
##                 3tri              56 ( 22.1%)    376 ( 36.6%)    432 ( 33.8%)
##                GA_ig               8 (  3.2%)     42 (  4.1%)     50 (  3.9%)
##                puerp             117 ( 46.2%)    349 ( 34.0%)    466 ( 36.4%)
##                Total             253 (100.0%)   1026 (100.0%)   1279 (100.0%)
## -------------------- ------- -------------- --------------- ---------------
##
## ----------------------------
##  Chi.squared   df   p.value
## ------------- ---- ---------
##    36.628      4       0
## ----------------------------
```

Comorbities

```r
#Cardiac
data7 <-  data7 %>%
  mutate(cardiac = case_when(CARDIOPATI == 1 ~ "yes",
                             CARDIOPATI == 2 ~ "no",
                             TRUE ~ NA_character_))
```

```
#Hematologic
data7 <-  data7 %>%
  mutate(hematologic = case_when(HEMATOLOGI == 1 ~ "yes",
                                 HEMATOLOGI == 2 ~ "no",
                                 TRUE ~ NA_character_))

#Hepatic
data7 <-  data7 %>%
  mutate(hepatic = case_when(HEPATICA == 1 ~ "yes",
                             HEPATICA == 2 ~ "no",
                             TRUE ~ NA_character_))

#Asthma
data7 <-  data7 %>%
  mutate(asthma = case_when(ASMA == 1 ~ "yes",
                            ASMA == 2 ~ "no",
                            TRUE ~ NA_character_))

#Diabetes
data7 <-  data7 %>%
  mutate(diabetes = case_when(DIABETES == 1 ~ "yes",
                              DIABETES == 2 ~ "no",
                              TRUE ~ NA_character_))

#Neurologic
data7 <-  data7 %>%
  mutate(neurologic = case_when(NEUROLOGIC == 1 ~ "yes",
                                NEUROLOGIC == 2 ~ "no",
                                TRUE ~ NA_character_))

#Pneumologic
data7 <-  data7 %>%
  mutate(pneumologic = case_when(PNEUMOPATI == 1 ~ "yes",
                                 PNEUMOPATI == 2 ~ "no",
                                 TRUE ~ NA_character_))

#Imunossupression
data7 <-  data7 %>%
  mutate(imuno = case_when(IMUNODEPRE == 1 ~ "yes",
                           IMUNODEPRE == 2 ~ "no",
                           TRUE ~ NA_character_))

#Renal
data7 <-  data7 %>%
  mutate(renal = case_when(RENAL == 1 ~ "yes",
                           RENAL == 2 ~ "no",
                           TRUE ~ NA_character_))

#Obesity
data7 <-  data7 %>%
  mutate(obesity = case_when(OBESIDADE == 1 ~ "yes",
                             OBESIDADE == 2 ~ "no",
                             TRUE ~ NA_character_))
```

```r
#Any comorbidity

df <- data7 %>%
  select(cardiac,obesity,hematologic,hepatic,asthma,diabetes,neurologic,pneumologic,imuno,renal)

#if all comorbities in df are NA (not available), return NA.
soma <- function(x){
  if (sum(is.na(x))==10)
    return(NA_character_)
  else
    return(sum(!is.na(x) & x=="yes"))
}
data7$qt_comorb_aux <- apply(df,1,soma)

data7 <- data7 %>%
  mutate(comorbidity = case_when(qt_comorb_aux >= 1 ~ "yes",
                                 qt_comorb_aux == 0 ~ "no",
                                 TRUE ~ NA_character_))
```

Cardiac

```r
with(data7, ctable(group, cardiac, prop = "r", useNA = "no", chisq = TRUE, OR = TRUE))
```

```
## Cross-Tabulation, Row Proportions
## group * cardiac
## Data Frame: data7
##
##
## ------------- --------- ------------- ------------- --------------
##                 cardiac            no           yes          Total
##         group
##   unspecified               98 (69.5%)    43 (30.5%)   141 (100.0%)
##      covid-19              411 (77.7%)   118 (22.3%)   529 (100.0%)
##         Total              509 (76.0%)   161 (24.0%)   670 (100.0%)
## ------------- --------- ------------- ------------- --------------
##
## ----------------------------
##  Chi.squared   df   p.value
## ------------- ---- ---------
##    3.6543      1    0.0559
## ----------------------------
##
## ----------------------------------
##  Odds Ratio   Lo - 95%   Hi - 95%
## ------------ ---------- ----------
##     0.65        0.43       0.99
## ----------------------------------
```

Hematologic

```
with(data7, ctable(group, hematologic, prop = "r", useNA = "no", chisq = FALSE, OR = TRUE))
```

```
## Cross-Tabulation, Row Proportions
## group * hematologic
## Data Frame: data7
##
##
## ------------- ------------- ------------- ----------- --------------
##                 hematologic            no         yes          Total
##         group
##   unspecified                 122 (95.3%)    6 (4.7%)   128 (100.0%)
##      covid-19                 495 (97.4%)   13 (2.6%)   508 (100.0%)
##         Total                 617 (97.0%)   19 (3.0%)   636 (100.0%)
## ------------- ------------- ------------- ----------- --------------
##
## ----------------------------------
##  Odds Ratio   Lo - 95%   Hi - 95%
## ------------ ---------- ----------
##     0.53        0.20       1.43
## ----------------------------------
```

```
with(data7, fisher.test(hematologic, group))
```

```
##
##  Fisher's Exact Test for Count Data
##
## data:  hematologic and group
## p-value = 0.2413
## alternative hypothesis: true odds ratio is not equal to 1
## 95 percent confidence interval:
##  0.1849903 1.7512422
## sample estimates:
## odds ratio
##  0.5346463
```

Diabetes

```
ctable(data7$group, data7$diabetes, chisq=TRUE, prop="r",  useNA = "no", OR = TRUE)
```

```
## Cross-Tabulation, Row Proportions
## group * diabetes
## Data Frame: data7
##
##
## ------------- ---------- ------------- ------------- --------------
##                 diabetes            no           yes          Total
##         group
```

```
##   unspecified              112 (82.4%)    24 (17.6%)   136 (100.0%)
##      covid-19              417 (78.1%)   117 (21.9%)   534 (100.0%)
##         Total              529 (79.0%)   141 (21.0%)   670 (100.0%)
## ------------- ---------- ------------- ------------- --------------
##
## ----------------------------
##  Chi.squared   df   p.value
## ------------- ---- ---------
##    0.9429      1    0.3315
## ----------------------------
##
## ----------------------------------
##  Odds Ratio   Lo - 95%   Hi - 95%
## ------------ ---------- ----------
##     1.31        0.81       2.13
## ----------------------------------
```

Obesity

```
ctable(data7$group, data7$obesity, chisq=TRUE, prop="r",  useNA = "no", OR = TRUE)
```

```
## Cross-Tabulation, Row Proportions
## group * obesity
## Data Frame: data7
##
##
## ------------- --------- ------------- ------------- --------------
##                 obesity            no           yes          Total
##         group
##   unspecified             114 (89.1%)    14 (10.9%)   128 (100.0%)
##      covid-19             405 (75.8%)   129 (24.2%)   534 (100.0%)
##         Total             519 (78.4%)   143 (21.6%)   662 (100.0%)
## ------------- --------- ------------- ------------- --------------
##
## ----------------------------
##  Chi.squared   df   p.value
## ------------- ---- ---------
##    9.8887      1    0.0017
## ----------------------------
##
## ----------------------------------
##  Odds Ratio   Lo - 95%   Hi - 95%
## ------------ ---------- ----------
##     2.59        1.44       4.68
## ----------------------------------
```

Asthma

```
ctable(data7$group, data7$asthma, chisq=TRUE, prop="r",  useNA = "no", OR = TRUE)
```

```
## Cross-Tabulation, Row Proportions
## group * asthma
## Data Frame: data7
##
##
## ------------- --------- ------------- ----------- --------------
##                  asthma            no         yes          Total
##         group
##   unspecified              122 (93.8%)    8 (6.2%)   130 (100.0%)
##      covid-19              470 (91.6%)   43 (8.4%)   513 (100.0%)
##         Total              592 (92.1%)   51 (7.9%)   643 (100.0%)
## ------------- --------- ------------- ----------- --------------
##
## ----------------------------
##  Chi.squared   df   p.value
## ------------- ---- ---------
##     0.433      1    0.5105
## ----------------------------
##
## ----------------------------------
##  Odds Ratio   Lo - 95%   Hi - 95%
## ------------ ---------- ----------
##     1.40        0.64       3.05
## ----------------------------------
```

Hepatic

```
ctable(data7$group, data7$hepatic, chisq=FALSE, prop="r",  useNA = "no", OR = TRUE)
```

```
## Cross-Tabulation, Row Proportions
## group * hepatic
## Data Frame: data7
##
##
## ------------- ---------- ------------- ----------- --------------
##                  hepatic            no         yes          Total
##         group
##   unspecified               126 (97.7%)    3 (2.3%)   129 (100.0%)
##      covid-19               495 (98.6%)    7 (1.4%)   502 (100.0%)
##         Total               621 (98.4%)   10 (1.6%)   631 (100.0%)
## ------------- ---------- ------------- ----------- --------------
##
## ----------------------------------
##  Odds Ratio   Lo - 95%   Hi - 95%
## ------------ ---------- ----------
##     0.59        0.15       2.33
## ----------------------------------
```

```
with(data7, fisher.test(hepatic, group))
```

```
##
```

```
##  Fisher's Exact Test for Count Data
##
## data:  hepatic and group
## p-value = 0.4349
## alternative hypothesis: true odds ratio is not equal to 1
## 95 percent confidence interval:
##  0.1333907 3.6125208
## sample estimates:
## odds ratio
##  0.5945033
```

Neurologic

```
ctable(data7$group, data7$neurologic, chisq=FALSE, prop="r",  useNA = "no", OR = TRUE)
```

```
## Cross-Tabulation, Row Proportions
## group * neurologic
## Data Frame: data7
##
##
## ------------- ------------ ------------- ----------- --------------
##                 neurologic            no         yes          Total
##         group
##   unspecified                123 (96.1%)    5 (3.9%)   128 (100.0%)
##      covid-19                496 (98.8%)    6 (1.2%)   502 (100.0%)
##         Total                619 (98.3%)   11 (1.7%)   630 (100.0%)
## ------------- ------------ ------------- ----------- --------------
##
## ----------------------------------
##  Odds Ratio   Lo - 95%   Hi - 95%
## ------------ ---------- ----------
##    0.298       0.089      0.991
## ----------------------------------
```

```
with(data7, fisher.test(neurologic, group))
```

```
##
##  Fisher's Exact Test for Count Data
##
## data:  neurologic and group
## p-value = 0.05196
## alternative hypothesis: true odds ratio is not equal to 1
## 95 percent confidence interval:
##  0.07447728 1.25717600
## sample estimates:
## odds ratio
##  0.2983128
```

Pneumologic

```
ctable(data7$group, data7$pneumologic, chisq=TRUE, prop="r",  useNA = "no", OR = TRUE)
```

```
## Cross-Tabulation, Row Proportions
## group * pneumologic
## Data Frame: data7
##
##
## ------------- ------------- ------------- ----------- --------------
##                 pneumologic            no         yes          Total
##         group
##   unspecified                 119 (93.0%)    9 (7.0%)   128 (100.0%)
##      covid-19                 493 (98.0%)   10 (2.0%)   503 (100.0%)
##         Total                 612 (97.0%)   19 (3.0%)   631 (100.0%)
## ------------- ------------- ------------- ----------- --------------
##
## ----------------------------
##  Chi.squared   df   p.value
## ------------- ---- ---------
##    7.2431      1    0.0071
## ----------------------------
##
## ----------------------------------
##  Odds Ratio   Lo - 95%   Hi - 95%
## ------------ ---------- ----------
##     0.27        0.11       0.67
## ----------------------------------
```

Imunossupression

```
ctable(data7$group, data7$imuno, chisq=TRUE, prop="r", useNA = "no", OR = TRUE)
```

```
## Cross-Tabulation, Row Proportions
## group * imuno
## Data Frame: data7
##
##
## ------------- ------- ------------- ----------- --------------
##                 imuno            no         yes          Total
##         group
##   unspecified           122 (94.6%)    7 (5.4%)   129 (100.0%)
##      covid-19           484 (95.7%)   22 (4.3%)   506 (100.0%)
##         Total           606 (95.4%)   29 (4.6%)   635 (100.0%)
## ------------- ------- ------------- ----------- --------------
##
## ----------------------------
##  Chi.squared   df   p.value
## ------------- ---- ---------
##    0.0827      1    0.7737
```

```
## ----------------------------
##
## ----------------------------------
##  Odds Ratio   Lo - 95%   Hi - 95%
## ------------ ---------- ----------
##     0.79        0.33       1.90
## ----------------------------------
```

Renal

```
ctable(data7$group, data7$renal, chisq=FALSE, prop="r",  useNA = "no", OR = TRUE)
```

```
## Cross-Tabulation, Row Proportions
## group * renal
## Data Frame: data7
##
##
## ------------- ------- ------------- ----------- --------------
##                 renal            no         yes          Total
##         group
##   unspecified           124 (96.1%)     5 (3.9%)   129 (100.0%)
##      covid-19           485 (97.4%)   13 (2.6%)   498 (100.0%)
##         Total           609 (97.1%)   18 (2.9%)   627 (100.0%)
## ------------- ------- ------------- ----------- --------------
##
## ----------------------------------
##  Odds Ratio   Lo - 95%   Hi - 95%
## ------------ ---------- ----------
##     0.66        0.23       1.90
## ----------------------------------
```

```
with(data7, fisher.test(renal, group))
```

```
##
##  Fisher's Exact Test for Count Data
##
## data:  renal and group
## p-value = 0.3904
## alternative hypothesis: true odds ratio is not equal to 1
## 95 percent confidence interval:
##  0.2174105 2.4288411
## sample estimates:
## odds ratio
##  0.6652248
```

Any comorbidity

```
with(data7, ctable(group, comorbidity, prop = "r", useNA = "no", chisq = TRUE, OR = TRUE))
```

```
## Cross-Tabulation, Row Proportions
## group * comorbidity
## Data Frame: data7
##
##
## ------------- ------------- ------------- ------------- --------------
##                 comorbidity            no           yes          Total
##         group
##   unspecified                  70 (44.6%)    87 (55.4%)   157 (100.0%)
##      covid-19                 272 (43.9%)   347 (56.1%)   619 (100.0%)
##         Total                 342 (44.1%)   434 (55.9%)   776 (100.0%)
## ------------- ------------- ------------- ------------- --------------
##
## ----------------------------
##  Chi.squared   df   p.value
## ------------- ---- ---------
##     0.003      1     0.956
## ----------------------------
##
## ----------------------------------
##  Odds Ratio   Lo - 95%   Hi - 95%
## ------------ ---------- ----------
##     1.03        0.72       1.46
## ----------------------------------
```

Symptoms

```
# Fever
data7 <-  data7 %>%
  mutate(fever = case_when(FEBRE == 1 ~ "yes",
                           FEBRE == 2 ~ "no",
                           TRUE ~ NA_character_))

# Cough
data7 <-  data7 %>%
  mutate(cough = case_when(TOSSE == 1 ~ "yes",
                           TOSSE == 2 ~ "no",
                           TRUE ~ NA_character_))

# Sore throat
data7 <-  data7 %>%
  mutate(sore_throat = case_when(GARGANTA == 1 ~ "yes",
                                 GARGANTA == 2 ~ "no",
                                 TRUE ~ NA_character_))

# Dyspnea
data7 <-  data7 %>%
  mutate(dyspnea = case_when(DISPNEIA == 1 ~ "yes",
                             DISPNEIA == 2 ~ "no",
                             TRUE ~ NA_character_))
```

```r
# Respiratory discomfort
data7 <-  data7 %>%
  mutate(resp_disc = case_when(DESC_RESP == 1 ~ "yes",
                               DESC_RESP == 2 ~ "no",
                               TRUE ~ NA_character_))

# Desaturation
data7 <-  data7 %>%
  mutate(desaturation = case_when(SATURACAO == 1 ~ "yes",
                                  SATURACAO == 2 ~ "no",
                                  TRUE ~ NA_character_))

# Diarrhea
data7 <-  data7 %>%
  mutate(diarrhea = case_when(DIARREIA == 1 ~ "yes",
                              DIARREIA == 2 ~ "no",
                              TRUE ~ NA_character_))

# Vomit
data7 <-  data7 %>%
  mutate(vomit = case_when(VOMITO == 1 ~ "yes",
                           VOMITO == 2 ~ "no",
                           TRUE ~ NA_character_))

# Abdominal pain
data7 <-  data7 %>%
  mutate(abd_pain = case_when(DOR_ABD == 1 ~ "yes",
                              DOR_ABD == 2 ~ "no",
                              TRUE ~ NA_character_))

# Fatigue
data7 <-  data7 %>%
  mutate(fatigue = case_when(FADIGA == 1 ~ "yes",
                             FADIGA == 2 ~ "no",
                             TRUE ~ NA_character_))

# Olfactory loss
data7 <-  data7 %>%
  mutate(olfac_loss = case_when(PERD_OLFT == 1 ~ "yes",
                                PERD_OLFT == 2 ~ "no",
                                TRUE ~ NA_character_))

# Loss of taste
data7 <-  data7 %>%
  mutate(loss_taste = case_when(PERD_PALA == 1 ~ "yes",
                                PERD_PALA == 2 ~ "no",
                                TRUE ~ NA_character_))

# Any respiratory symptom
df <- data7 %>%
  select(dyspnea,fatigue,desaturation,resp_disc)

soma <- function(x){
```

```r
  if (sum(is.na(x))==4)
    return(NA_character_)
  else
    return(sum(!is.na(x) & x=="yes"))
}
data7$qt_sintomas_resp_aux <- apply(df,1,soma)

data7 <-  data7 %>%
  mutate(resp_symp = case_when(qt_sintomas_resp_aux >=1 ~ "yes",
                               qt_sintomas_resp_aux ==0 ~ "no",
                               TRUE ~ NA_character_))
# Any symptom
df <- data7 %>%
  select(dyspnea,fatigue,desaturation,resp_disc,
         fever,cough,sore_throat,diarrhea,vomit,abd_pain,olfac_loss,loss_taste)
soma <- function(x){
  if (sum(is.na(x))==12)
    return(NA_character_)
  else
    return(sum(!is.na(x) & x=="yes"))
}
data7$qt_sintomas_aux <- apply(df,1,soma)

data7 <-  data7 %>%
  mutate(symptom = case_when(qt_sintomas_aux >= 1 ~ "yes",
                             qt_sintomas_aux == 0 ~ "no",
                             TRUE ~ NA_character_))
```

Fever

```r
with(data7, ctable(group, fever, prop = "r", useNA = "no", chisq = TRUE, OR=TRUE))
```

```
## Cross-Tabulation, Row Proportions
## group * fever
## Data Frame: data7
##
##
## ------------- ------- ------------- ------------- ---------------
##                 fever            no           yes           Total
##         group
##   unspecified             90 (44.1%)   114 (55.9%)    204 (100.0%)
##      covid-19            276 (31.1%)   611 (68.9%)    887 (100.0%)
##         Total            366 (33.5%)   725 (66.5%)   1091 (100.0%)
## ------------- ------- ------------- ------------- ---------------
##
## ----------------------------
##  Chi.squared   df   p.value
## ------------- ---- ---------
##    11.9997     1     5e-04
## ----------------------------
##
```

```
## ----------------------------------
##  Odds Ratio   Lo - 95%   Hi - 95%
## ------------ ---------- ----------
##     1.75        1.28       2.38
## ----------------------------------
```

Cough

```r
with(data7, ctable(group, cough, prop = "r", useNA = "no", chisq = TRUE, OR=TRUE))
```

```
## Cross-Tabulation, Row Proportions
## group * cough
## Data Frame: data7
##
##
## ------------- ------- ------------- ------------- ---------------
##                 cough            no           yes           Total
##         group
##   unspecified             89 (42.2%)   122 (57.8%)    211 (100.0%)
##      covid-19            189 (20.6%)   729 (79.4%)    918 (100.0%)
##         Total            278 (24.6%)   851 (75.4%)   1129 (100.0%)
## ------------- ------- ------------- ------------- ---------------
##
## ----------------------------
##  Chi.squared   df   p.value
## ------------- ---- ---------
##    41.9393     1       0
## ----------------------------
##
## ----------------------------------
##  Odds Ratio   Lo - 95%   Hi - 95%
## ------------ ---------- ----------
##     2.81        2.05       3.86
## ----------------------------------
```

Sore throat

```r
with(data7, ctable(group, sore_throat, prop = "r", useNA = "no", chisq = TRUE, OR=TRUE))
```

```
## Cross-Tabulation, Row Proportions
## group * sore_throat
## Data Frame: data7
##
##
## ------------- ------------- ------------- ------------- --------------
##                 sore_throat            no           yes          Total
##         group
##   unspecified                 146 (81.6%)    33 (18.4%)   179 (100.0%)
##      covid-19                 557 (74.3%)   193 (25.7%)   750 (100.0%)
```

```
##         Total                 703 (75.7%)   226 (24.3%)   929 (100.0%)
## ------------- ------------- ------------- ------------- --------------
##
## ----------------------------
##  Chi.squared   df   p.value
## ------------- ---- ---------
##    3.7934      1    0.0515
## ----------------------------
##
## ----------------------------------
##  Odds Ratio   Lo - 95%   Hi - 95%
## ------------ ---------- ----------
##     1.53        1.02       2.31
## ----------------------------------
```

Dyspnea

```
with(data7, ctable(group, dyspnea, prop = "r", useNA = "no", chisq = TRUE, OR=TRUE))
```

```
## Cross-Tabulation, Row Proportions
## group * dyspnea
## Data Frame: data7
##
##
## ------------- --------- ------------- ------------- ---------------
##                 dyspnea            no           yes           Total
##         group
##   unspecified               34 (15.1%)   191 (84.9%)    225 (100.0%)
##      covid-19              132 (14.1%)   807 (85.9%)    939 (100.0%)
##         Total              166 (14.3%)   998 (85.7%)   1164 (100.0%)
## ------------- --------- ------------- ------------- ---------------
##
## ----------------------------
##  Chi.squared   df   p.value
## ------------- ---- ---------
##    0.0899      1    0.7643
## ----------------------------
##
## ----------------------------------
##  Odds Ratio   Lo - 95%   Hi - 95%
## ------------ ---------- ----------
##     1.09        0.72       1.64
## ----------------------------------
```

Respiratory discomfort

```
with(data7, ctable(group, resp_disc, prop = "r", useNA = "no", chisq = TRUE, OR=TRUE))
```

```
## Cross-Tabulation, Row Proportions
```

```
## group * resp_disc
## Data Frame: data7
##
##
## ------------- ----------- ------------- ------------- ---------------
##                 resp_disc            no           yes           Total
##         group
##   unspecified                46 (21.0%)   173 (79.0%)    219 (100.0%)
##      covid-19               203 (22.7%)   690 (77.3%)    893 (100.0%)
##         Total               249 (22.4%)   863 (77.6%)   1112 (100.0%)
## ------------- ----------- ------------- ------------- ---------------
##
## ----------------------------
##  Chi.squared   df   p.value
## ------------- ---- ---------
##    0.2109      1    0.6461
## ----------------------------
##
## ----------------------------------
##  Odds Ratio   Lo - 95%   Hi - 95%
## ------------ ---------- ----------
##     0.90        0.63       1.30
## ----------------------------------
```

Desaturation

```
with(data7, ctable(group, desaturation, prop = "r", useNA = "no", chisq = TRUE, OR=TRUE))
```

```
## Cross-Tabulation, Row Proportions
## group * desaturation
## Data Frame: data7
##
##
## ------------- -------------- ------------- ------------- ---------------
##                 desaturation            no           yes           Total
##         group
##   unspecified                   58 (27.5%)   153 (72.5%)    211 (100.0%)
##      covid-19                  195 (21.9%)   695 (78.1%)    890 (100.0%)
##         Total                  253 (23.0%)   848 (77.0%)   1101 (100.0%)
## ------------- -------------- ------------- ------------- ---------------
##
## ----------------------------
##  Chi.squared   df   p.value
## ------------- ---- ---------
##    2.6916      1    0.1009
## ----------------------------
##
## ----------------------------------
##  Odds Ratio   Lo - 95%   Hi - 95%
## ------------ ---------- ----------
##     1.35        0.96       1.90
## ----------------------------------
```

Diarrhea

```r
with(data7, ctable(group, diarrhea, prop = "r", useNA = "no", chisq = TRUE, OR=TRUE))
```

```
## Cross-Tabulation, Row Proportions
## group * diarrhea
## Data Frame: data7
##
##
## ------------- ---------- ------------- ------------- --------------
##                 diarrhea            no           yes          Total
##         group
##   unspecified              158 (88.8%)    20 (11.2%)   178 (100.0%)
##      covid-19              631 (85.2%)   110 (14.8%)   741 (100.0%)
##         Total              789 (85.9%)   130 (14.1%)   919 (100.0%)
## ------------- ---------- ------------- ------------- --------------
##
## ----------------------------
##  Chi.squared   df   p.value
## ------------- ---- ---------
##    1.2563      1    0.2624
## ----------------------------
##
## ----------------------------------
##  Odds Ratio   Lo - 95%   Hi - 95%
## ------------ ---------- ----------
##     1.38        0.83       2.29
## ----------------------------------
```

Vomit

```r
with(data7, ctable(group, vomit, prop = "r", useNA = "no", chisq = TRUE, OR=TRUE))
```

```
## Cross-Tabulation, Row Proportions
## group * vomit
## Data Frame: data7
##
##
## ------------- ------- ------------- ------------- --------------
##                 vomit            no           yes          Total
##         group
##   unspecified           150 (82.0%)    33 (18.0%)   183 (100.0%)
##      covid-19           642 (87.6%)    91 (12.4%)   733 (100.0%)
##         Total           792 (86.5%)   124 (13.5%)   916 (100.0%)
## ------------- ------- ------------- ------------- --------------
##
## ----------------------------
##  Chi.squared   df   p.value
## ------------- ---- ---------
##    3.4835      1     0.062
```

```
## ----------------------------
##
## ----------------------------------
##  Odds Ratio   Lo - 95%   Hi - 95%
## ------------ ---------- ----------
##     0.64        0.42       1.00
## ----------------------------------
```

Abdominal pain

```
with(data7, ctable(group, abd_pain, prop = "r", useNA = "no", chisq = TRUE, OR=TRUE))
```

```
## Cross-Tabulation, Row Proportions
## group * abd_pain
## Data Frame: data7
##
##
## ------------- ---------- ------------- ------------ --------------
##                 abd_pain            no          yes          Total
##         group
##   unspecified               88 (89.8%)   10 (10.2%)    98 (100.0%)
##      covid-19              495 (89.4%)   59 (10.6%)   554 (100.0%)
##         Total              583 (89.4%)   69 (10.6%)   652 (100.0%)
## ------------- ---------- ------------- ------------ --------------
##
## ----------------------------
##  Chi.squared   df   p.value
## ------------- ---- ---------
##       0        1       1
## ----------------------------
##
## ----------------------------------
##  Odds Ratio   Lo - 95%   Hi - 95%
## ------------ ---------- ----------
##     1.05        0.52       2.13
## ----------------------------------
```

Fatigue

```
with(data7, ctable(group, fatigue, prop = "r", useNA = "no", chisq = TRUE, OR=TRUE))
```

```
## Cross-Tabulation, Row Proportions
## group * fatigue
## Data Frame: data7
##
##
## ------------- --------- ------------- ------------- --------------
##                 fatigue            no           yes          Total
##         group
```

```
##   unspecified              79 (80.6%)    19 (19.4%)    98 (100.0%)
##      covid-19             368 (64.3%)   204 (35.7%)   572 (100.0%)
##         Total             447 (66.7%)   223 (33.3%)   670 (100.0%)
## ------------- --------- ------------- ------------- --------------
##
## ----------------------------
##  Chi.squared   df   p.value
## ------------- ---- ---------
##    9.2623      1    0.0023
## ----------------------------
##
## ----------------------------------
##  Odds Ratio   Lo - 95%   Hi - 95%
## ------------ ---------- ----------
##     2.30        1.36       3.91
## ----------------------------------
```

Olfactory loss

```
with(data7, ctable(group, olfac_loss, prop = "r", useNA = "no", chisq = FALSE, OR=TRUE))
```

```
## Cross-Tabulation, Row Proportions
## group * olfac_loss
## Data Frame: data7
##
##
## ------------- ------------ ------------- ------------ --------------
##                 olfac_loss            no          yes          Total
##         group
##   unspecified                 91 (94.8%)    5 ( 5.2%)    96 (100.0%)
##      covid-19                470 (83.6%)   92 (16.4%)   562 (100.0%)
##         Total                561 (85.3%)   97 (14.7%)   658 (100.0%)
## ------------- ------------ ------------- ------------ --------------
##
## ----------------------------------
##  Odds Ratio   Lo - 95%   Hi - 95%
## ------------ ---------- ----------
##     3.56        1.41       9.01
## ----------------------------------
```

```
with(data7, fisher.test(olfac_loss, group))
```

```
##
##  Fisher's Exact Test for Count Data
##
## data:  olfac_loss and group
## p-value = 0.002827
## alternative hypothesis: true odds ratio is not equal to 1
## 95 percent confidence interval:
##   1.41211 11.52657
## sample estimates:
```

```
## odds ratio
##   3.557888
```

Loss of taste

```
with(data7, ctable(group, loss_taste, prop = "r", useNA = "no", chisq = FALSE, OR=TRUE))
```

```
## Cross-Tabulation, Row Proportions
## group * loss_taste
## Data Frame: data7
##
##
## ------------- ------------ ------------- ------------ --------------
##                 loss_taste            no          yes          Total
##         group
##   unspecified                  91 (94.8%)    5 ( 5.2%)    96 (100.0%)
##      covid-19                 478 (84.8%)   86 (15.2%)   564 (100.0%)
##         Total                 569 (86.2%)   91 (13.8%)   660 (100.0%)
## ------------- ------------ ------------- ------------ --------------
##
## ----------------------------------
##  Odds Ratio   Lo - 95%   Hi - 95%
## ------------ ---------- ----------
##     3.27        1.29       8.29
## ----------------------------------
```

```
with(data7, fisher.test(loss_taste, group))
```

```
##
##  Fisher's Exact Test for Count Data
##
## data:  loss_taste and group
## p-value = 0.006158
## alternative hypothesis: true odds ratio is not equal to 1
## 95 percent confidence interval:
##   1.295082 10.613606
## sample estimates:
## odds ratio
##   3.270412
```

Any respiratory symptom

```
with(data7, ctable(group, resp_symp, prop = "r", useNA = "no", chisq = TRUE, OR=TRUE))
```

```
## Cross-Tabulation, Row Proportions
## group * resp_symp
## Data Frame: data7
##
```

```
##
## ------------- ----------- ----------- -------------- ---------------
##                 resp_symp          no            yes           Total
##         group
##   unspecified               18 (7.5%)    221 (92.5%)    239 (100.0%)
##      covid-19               49 (5.0%)    935 (95.0%)    984 (100.0%)
##         Total               67 (5.5%)   1156 (94.5%)   1223 (100.0%)
## ------------- ----------- ----------- -------------- ---------------
##
## ----------------------------
##  Chi.squared   df   p.value
## ------------- ---- ---------
##    1.9503      1    0.1626
## ----------------------------
##
## ----------------------------------
##  Odds Ratio   Lo - 95%   Hi - 95%
## ------------ ---------- ----------
##     1.55        0.89       2.72
## ----------------------------------
```

Any symptom

```
with(data7, ctable(group, symptom, prop = "r", useNA = "no", chisq = FALSE, OR=TRUE))
```

```
## Cross-Tabulation, Row Proportions
## group * symptom
## Data Frame: data7
##
##
## ------------- --------- ----------- -------------- ---------------
##                 symptom          no            yes           Total
##         group
##   unspecified              4 (1.6%)    242 (98.4%)    246 (100.0%)
##      covid-19             10 (1.0%)    998 (99.0%)   1008 (100.0%)
##         Total             14 (1.1%)   1240 (98.9%)   1254 (100.0%)
## ------------- --------- ----------- -------------- ---------------
##
## ----------------------------------
##  Odds Ratio   Lo - 95%   Hi - 95%
## ------------ ---------- ----------
##     1.65        0.51       5.30
## ----------------------------------
```

```
with(data7, fisher.test(symptom, group))
```

```
##
##  Fisher's Exact Test for Count Data
##
## data:  symptom and group
## p-value = 0.4942
```

```
## alternative hypothesis: true odds ratio is not equal to 1
## 95 percent confidence interval:
##  0.374257 5.778501
## sample estimates:
## odds ratio
##   1.648827
```

Outcome

```
# Hospital-acquired infection
data7 <-  data7 %>%
  mutate(hospital_infection = case_when(NOSOCOMIAL ==1  ~ "yes",
                           NOSOCOMIAL == 2 ~ "no",
                             TRUE ~ NA_character_))
# ICU
data7 <- data7 %>%
  mutate(icu = case_when(UTI == 1 ~ "yes",
                         UTI == 2 ~ "no",
                         TRUE ~ NA_character_))

# Intubation
data7 <- data7 %>%
  mutate(intubation = case_when(SUPORT_VEN == 1 ~ "yes",
                                SUPORT_VEN == 2 | SUPORT_VEN == 3 ~ "no",
                                TRUE ~ NA_character_))
```

Hospital-acquired infection

```
with(data7, ctable(group, hospital_infection, prop = "r", useNA = "no", chisq = TRUE, OR=TRUE))
```

```
## Cross-Tabulation, Row Proportions
## group * hospital_infection
## Data Frame: data7
##
##
## ------------- -------------------- ------------- ----------- --------------
##                  hospital_infection            no         yes          Total
##         group
##   unspecified                        169 (92.9%)   13 (7.1%)   182 (100.0%)
##      covid-19                        749 (96.8%)   25 (3.2%)   774 (100.0%)
##         Total                        918 (96.0%)   38 (4.0%)   956 (100.0%)
## ------------- -------------------- ------------- ----------- --------------
##
## ----------------------------
##  Chi.squared   df   p.value
## ------------- ---- ---------
##     4.93       1    0.0264
## ----------------------------
##
```

```
## -----------------------------------
##  Odds Ratio   Lo - 95%   Hi - 95%
## ------------ ---------- ----------
##     0.43        0.22       0.87
## -----------------------------------
```

ICU

```
with(data7, ctable(group, icu, prop = "r", useNA = "no", chisq = TRUE, OR=TRUE))
```

```
## Cross-Tabulation, Row Proportions
## group * icu
## Data Frame: data7
##
##
## ------------- ----- ------------- ------------- ---------------
##                 icu            no           yes           Total
##         group
##   unspecified           69 (30.8%)   155 (69.2%)    224 (100.0%)
##      covid-19          207 (22.4%)   718 (77.6%)    925 (100.0%)
##         Total          276 (24.0%)   873 (76.0%)   1149 (100.0%)
## ------------- ----- ------------- ------------- ---------------
##
## ----------------------------
##  Chi.squared   df   p.value
## ------------- ---- ---------
##    6.5596      1    0.0104
## ----------------------------
##
## -----------------------------------
##  Odds Ratio   Lo - 95%   Hi - 95%
## ------------ ---------- ----------
##     1.54        1.12       2.13
## -----------------------------------
```

Duration of hospitalization in ICU

```
data7 <- data7 %>%
   dplyr::mutate(
    dt_entuti = as.Date(DT_ENTUTI, format = "%d/%m/%Y"),
    dt_saiduti = as.Date(DT_SAIDUTI, format = "%d/%m/%Y"),
    icu_days = as.numeric(dt_saiduti-dt_entuti)
)
```

```
datasummary((group) ~ icu_days*(n+media+DP+mediana+minimo+maximo+q25+q75+IQR),
            data = data7, output = 'markdown')
```

	n	media	DP	mediana	minimo	maximo	q25	q75	IQR
unspecified	80.00	7.70	12.88	3.00	0.00	70.00	1.00	8.25	7.25
covid-19	430.00	13.61	14.25	11.00	0.00	183.00	5.00	19.00	14.00

```
wilcox.test(icu_days ~ group, data = data7)
```

```
##
##  Wilcoxon rank sum test with continuity correction
##
## data:  icu_days by group
## W = 9665.5, p-value = 4.655e-10
## alternative hypothesis: true location shift is not equal to 0
```

Intubation

```
with(data7, ctable(group, intubation, prop = "r", useNA = "no", chisq = TRUE, OR=TRUE))
```

```
## Cross-Tabulation, Row Proportions
## group * intubation
## Data Frame: data7
##
##
## ------------- ------------ ------------- ------------- ---------------
##                 intubation            no           yes           Total
##         group
##   unspecified                 90 (40.0%)   135 (60.0%)    225 (100.0%)
##      covid-19                305 (33.4%)   608 (66.6%)    913 (100.0%)
##         Total                395 (34.7%)   743 (65.3%)   1138 (100.0%)
## ------------- ------------ ------------- ------------- ---------------
##
## ----------------------------
##  Chi.squared   df   p.value
## ------------- ---- ---------
##    3.1782      1    0.0746
## ----------------------------
##
## ----------------------------------
##  Odds Ratio   Lo - 95%   Hi - 95%
## ------------ ---------- ----------
##     1.33        0.98       1.79
## ----------------------------------
```

Time elapsed between the start of symptoms and the date of death

We will analyze the time between the onset of the first symptoms until the patient's death. Let's create a new variable that will count the number of days of this difference.

```r
# Creation of time between the start of symptoms and the date of death
data7 <- data7 %>%
   dplyr::mutate(
    dt_sin_pri = as.Date(DT_SIN_PRI, format = "%d/%m/%Y"),
    dt_evoluca = as.Date(DT_EVOLUCA, format = "%d/%m/%Y"),
    days_symp_death = as.numeric(dt_evoluca-dt_sin_pri)
)
```

```r
datasummary((group) ~ days_symp_death*(n+media+DP+mediana+minimo+maximo+q25+q75+IQR),
            data = data7, output = 'markdown')
```

	n	media	DP	mediana	minimo	maximo	q25	q75	IQR
unspecified	251.00	11.53	13.45	8.00	0.00	91.00	3.00	15.00	12.00
covid-19	1022.00	20.00	15.04	18.00	0.00	222.00	11.00	26.00	15.00

```r
wilcox.test(days_symp_death ~ group, data = data7)
```

```
##
##  Wilcoxon rank sum test with continuity correction
##
## data:  days_symp_death by group
## W = 67126, p-value < 2.2e-16
## alternative hypothesis: true location shift is not equal to 0
```

Distribution of COVID-19 and unspecified etiologic cause by Brazilian states

The distribution of COVID-19 and unspecified etiologic cause cases by Brazilian state (SG_UF variable) is presented.

```r
with(data7, ctable(SG_UF, group, prop = "c")) #SG_UF indicates Brazilian state
```

```
## Cross-Tabulation, Column Proportions
## SG_UF * group
## Data Frame: data7
##
## ------- ------- -------------- --------------- ----------------
##           group    unspecified        covid-19            Total
##   SG_UF
##      AC              0 (  0.0%)      2 (  0.2%)      2 (  0.16%)
##      AL              4 (  1.6%)     19 (  1.9%)     23 (  1.80%)
##      AM              6 (  2.4%)     67 (  6.5%)     73 (  5.71%)
##      AP              0 (  0.0%)      6 (  0.6%)      6 (  0.47%)
##      BA             25 (  9.9%)     49 (  4.8%)     74 (  5.79%)
##      CE              9 (  3.6%)     54 (  5.3%)     63 (  4.93%)
##      DF              1 (  0.4%)     16 (  1.6%)     17 (  1.33%)
##      ES              6 (  2.4%)     16 (  1.6%)     22 (  1.72%)
##      GO              8 (  3.2%)     58 (  5.7%)     66 (  5.16%)
```

```
##      MA              4 (  1.6%)     41 (  4.0%)     45 (  3.52%)
##      MG             22 (  8.7%)     78 (  7.6%)    100 (  7.82%)
##      MS              4 (  1.6%)     20 (  1.9%)     24 (  1.88%)
##      MT              5 (  2.0%)     14 (  1.4%)     19 (  1.49%)
##      PA             14 (  5.5%)     38 (  3.7%)     52 (  4.07%)
##      PB             12 (  4.7%)     22 (  2.1%)     34 (  2.66%)
##      PE             17 (  6.7%)     27 (  2.6%)     44 (  3.44%)
##      PI              0 (  0.0%)     12 (  1.2%)     12 (  0.94%)
##      PR             13 (  5.1%)     48 (  4.7%)     61 (  4.77%)
##      RJ             22 (  8.7%)    122 ( 11.9%)    144 ( 11.26%)
##      RN              5 (  2.0%)     24 (  2.3%)     29 (  2.27%)
##      RO              2 (  0.8%)     16 (  1.6%)     18 (  1.41%)
##      RR              0 (  0.0%)     18 (  1.8%)     18 (  1.41%)
##      RS              9 (  3.6%)     34 (  3.3%)     43 (  3.36%)
##      SC              3 (  1.2%)     19 (  1.9%)     22 (  1.72%)
##      SE              1 (  0.4%)      9 (  0.9%)     10 (  0.78%)
##      SP             60 ( 23.7%)    186 ( 18.1%)    246 ( 19.23%)
##      TO              1 (  0.4%)     10 (  1.0%)     11 (  0.86%)
##    <NA>              0 (  0.0%)      1 (  0.1%)      1 (  0.08%)
##   Total            253 (100.0%)   1026 (100.0%)   1279 (100.00%)
## ------- ------- -------------- --------------- ----------------
```

Now we consider the rate of deaths per 100,000 live births in each group. The live births data considered is from 2019 and it is available in the link on the link: http://svs.aids.gov.br/dantps/centrais-de-conteudos/paineis-de-monitoramento/natalidade/nascidos-vivos.

```r
# Database of live births in the year 2019 in Brazil
data_birth <- read_excel("dados_nascidos_2019.xlsx")
```

Covid-19 group

```r
#Covid-19
d1 <- data7 %>%
  filter(group == "covid-19")

valor <- data.frame(table(d1$SG_UF))
colnames(valor) <- c("uf", "n")

dt1_state <- left_join(valor, data_birth, by= "uf")

dt1_state <- dt1_state %>%
  mutate(T1 = (n/total)*100000)

states <- read_state(year = 2020)
```

```
##   |                                                                              |
```

```r
states <- dplyr::left_join(states, dt1_state, by = c("abbrev_state" = "uf"))

g1 <- ggplot(data= states) +
  geom_sf(aes(fill=T1), color= "grey30", size=.15) +
```

```
  theme_void()  +
  labs(fill = "Death \n rate",title="COVID-19 cases") +
  scale_fill_distiller(palette="Purples",trans="reverse")

g1
```

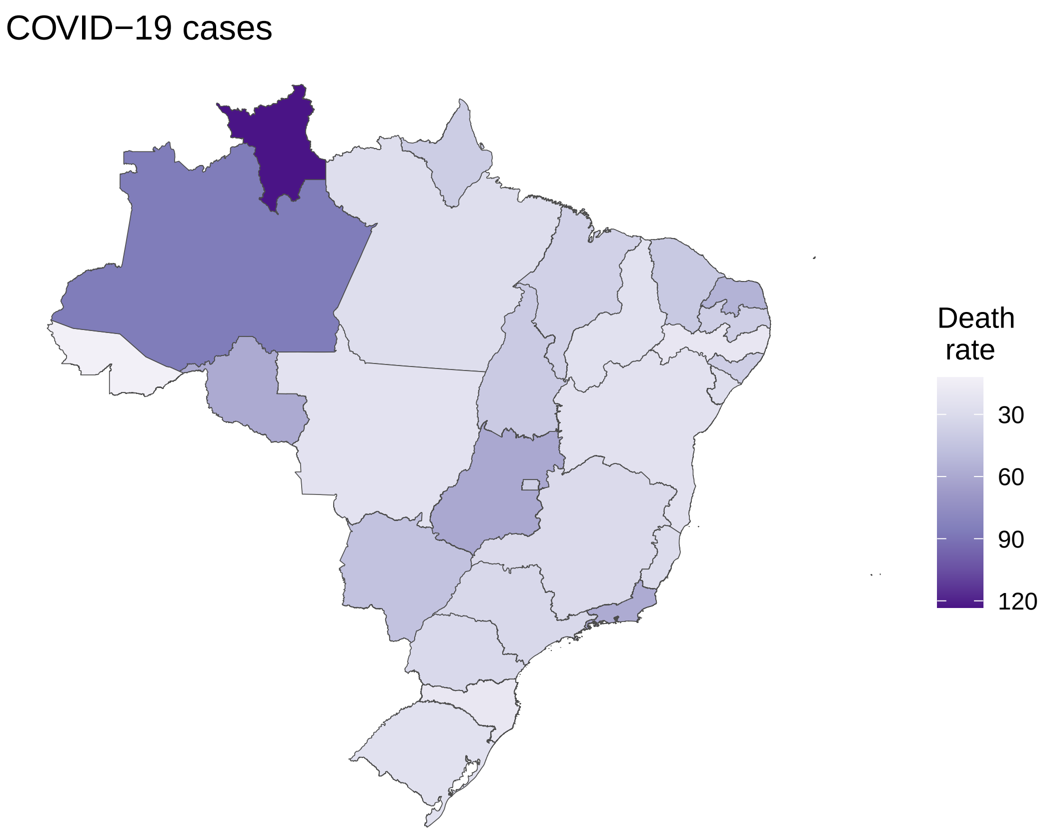


```
ggsave("covid-19_cases",dpi="print",device="tiff")
```

Unspecified cause

```
#Unspecified cause
d2 <- data7 %>%
  filter(group == "unspecified")

valor <- data.frame(table(d2$SG_UF))
colnames(valor) <- c("uf", "n")

dt2_state <- left_join(valor, data_birth, by= "uf")

dt2_state <- dt2_state %>%
  mutate(T1 = (n/total)*100000)

states2 <- read_state(year = 2020)
```

```
## |                                                                          |
```

```
states2 <- dplyr::left_join(states2, dt2_state, by = c("abbrev_state" = "uf"))

g2 <- ggplot(data= states2) +
  geom_sf(aes(fill=T1), color= "grey30", size=.15) +
  theme_void()  +
  labs(fill = "Death rate",title="Cases with unspecified cause")  +
  scale_fill_distiller(palette="Purples",trans="reverse")

g2
```

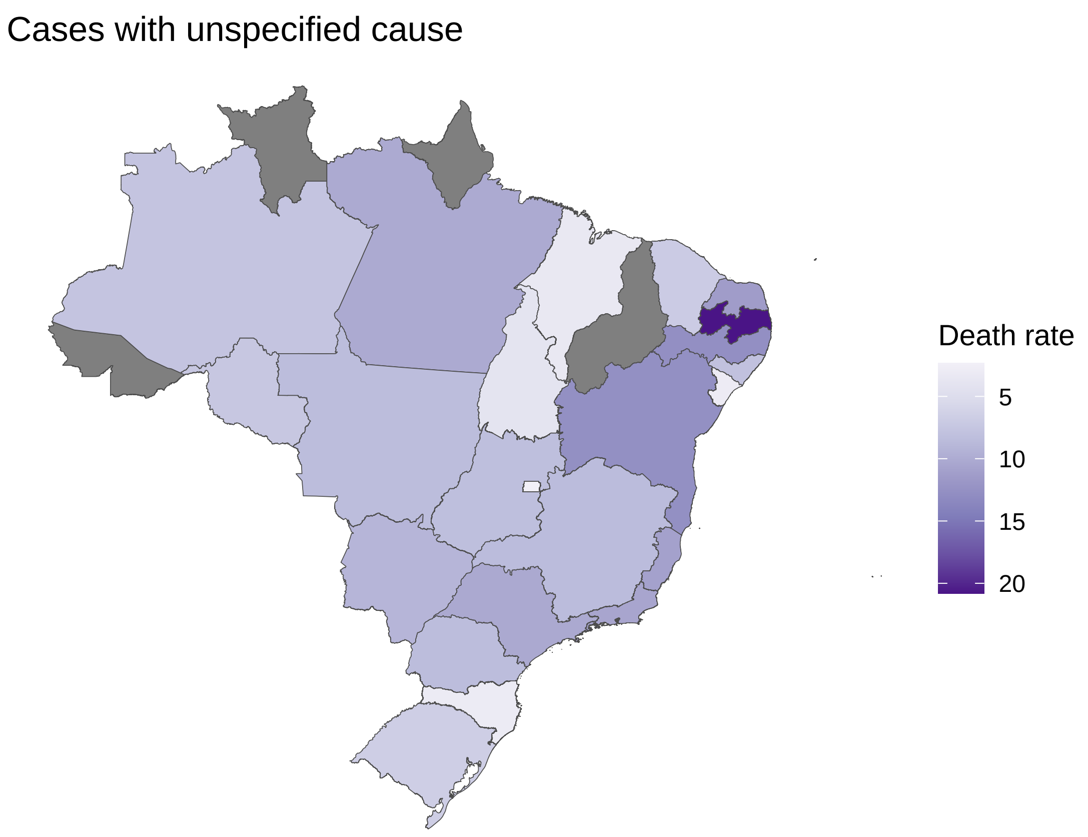


```
ggsave("unspecified_cases",dpi="print",device="tiff")
```

Distribution by Epidemiological Week

First, let's create the variable that will indicate the epidemiological week together with the year case.

```
data7 <- data7 %>%
  mutate(dt_sin_pri = as.Date(DT_SIN_PRI, format = "%d/%m/%Y")
)
data7 <-  data7 %>%
  mutate(seman_pri = paste(formatC(SEM_PRI, width=2, format="d", flag="0"),
```

```
                             year(dt_sin_pri),sep="/"))

d1 <- rownames_to_column(data.frame(freq(data7$seman_pri,  cum=FALSE,total=TRUE,na.last=FALSE,valid=FALS
aux <- str_split(d1$week,"/",simplify=TRUE)
d1<- data.frame(aux,d1)
d1$week<- NULL
d1 <- d1 %>% arrange(X2,X1)
d1<- d1[-c(1,2),-c(5,6,7)]
d1 <- rename(d1,"Week"="X1","Year"="X2","n"="Freq","%"="X..Valid")
```

```
#frequency table
d1
```

```
##    Week Year  n         %
## 3    08 2020  3 0.2345582
## 4    11 2020  4 0.3127443
## 5    12 2020  7 0.5473026
## 6    13 2020  6 0.4691165
## 7    14 2020 14 1.0946052
## 8    15 2020 17 1.3291634
## 9    16 2020 24 1.8764660
## 10   17 2020 35 2.7365129
## 11   18 2020 25 1.9546521
## 12   19 2020 39 3.0492572
## 13   20 2020 33 2.5801407
## 14   21 2020 21 1.6419077
## 15   22 2020 23 1.7982799
## 16   23 2020 26 2.0328382
## 17   24 2020 17 1.3291634
## 18   25 2020 29 2.2673964
## 19   26 2020 25 1.9546521
## 20   27 2020 25 1.9546521
## 21   28 2020 28 2.1892103
## 22   29 2020 16 1.2509773
## 23   30 2020 19 1.4855356
## 24   31 2020 15 1.1727912
## 25   32 2020 11 0.8600469
## 26   33 2020 12 0.9382330
## 27   34 2020 11 0.8600469
## 28   35 2020 10 0.7818608
## 29   36 2020  7 0.5473026
## 30   37 2020 11 0.8600469
## 31   38 2020 11 0.8600469
## 32   39 2020  5 0.3909304
## 33   40 2020  6 0.4691165
## 34   41 2020  5 0.3909304
## 35   42 2020  7 0.5473026
## 36   43 2020  5 0.3909304
## 37   44 2020  4 0.3127443
## 38   45 2020 13 1.0164191
## 39   46 2020 14 1.0946052
## 40   47 2020 14 1.0946052
## 41   48 2020  5 0.3909304
```

```
## 42   49 2020  7 0.5473026
## 43   50 2020 11 0.8600469
## 44   51 2020 14 1.0946052
## 45   52 2020 19 1.4855356
## 46   53 2020  6 0.4691165
## 47   01 2021 24 1.8764660
## 48   02 2021 14 1.0946052
## 49   03 2021 20 1.5637217
## 50   04 2021 21 1.6419077
## 51   05 2021 23 1.7982799
## 52   06 2021 25 1.9546521
## 53   07 2021 40 3.1274433
## 54   08 2021 52 4.0656763
## 55   09 2021 71 5.5512119
## 56   10 2021 84 6.5676310
## 57   11 2021 86 6.7240031
## 58   12 2021 56 4.3784206
## 59   13 2021 55 4.3002346
## 60   14 2021 32 2.5019547
## 61   15 2021 15 1.1727912
## 62   53 2021  2 0.1563722
```

Now, to better understand the distribution of time, let's make a graph referring to the month of the first symptom for better visualization. The graph takes into account the percentage of unspecified cases so that the complement is the percentage of covid-19 cases.

```
#FILTERING CASES UNTIL MARCH 2021
d1 <- data7[data7$dt_sin_pri < as.Date("01/04/2021",format="%d/%m/%Y"),]

d1 <- d1 %>%
  mutate(month_year = paste(formatC(month(dt_sin_pri), width=2, format="d", flag="0"),
                            year(dt_sin_pri),sep="/"))

d <- prop.table(table(d1$month_year,d1$group),1)
G4 <- as.data.frame(d)
G3 <- G4[G4$Var2 == "unspecified",]
G3$Freq <- round((G3$Freq)*100, 2)
Sys.setlocale("LC_TIME","C")
```

```
## [1] "C"
```

```
G3$Var1 <- as.yearmon(G3$Var1, format = "%m/%Y")
G3$Var3 <- as.Date(format(G3$Var1,"%Y-%m-01"))




ggplot(data=G3, aes(x = Var3, y=Freq)) +
  geom_line(size=1.2, color="indianred2") +
  geom_point(size=3,color="indianred2") +
  geom_hline(yintercept = 50) + xlab("Month of first symptom") +
  ylab("Relative frequency (%)") + scale_x_date(labels = date_format("%h %y"),
               breaks = seq(from = min(G3$Var3),
                            to = max(G3$Var3), by = "month")) +
  theme(axis.text.x = element_text(angle = 60, hjust = 1))
```

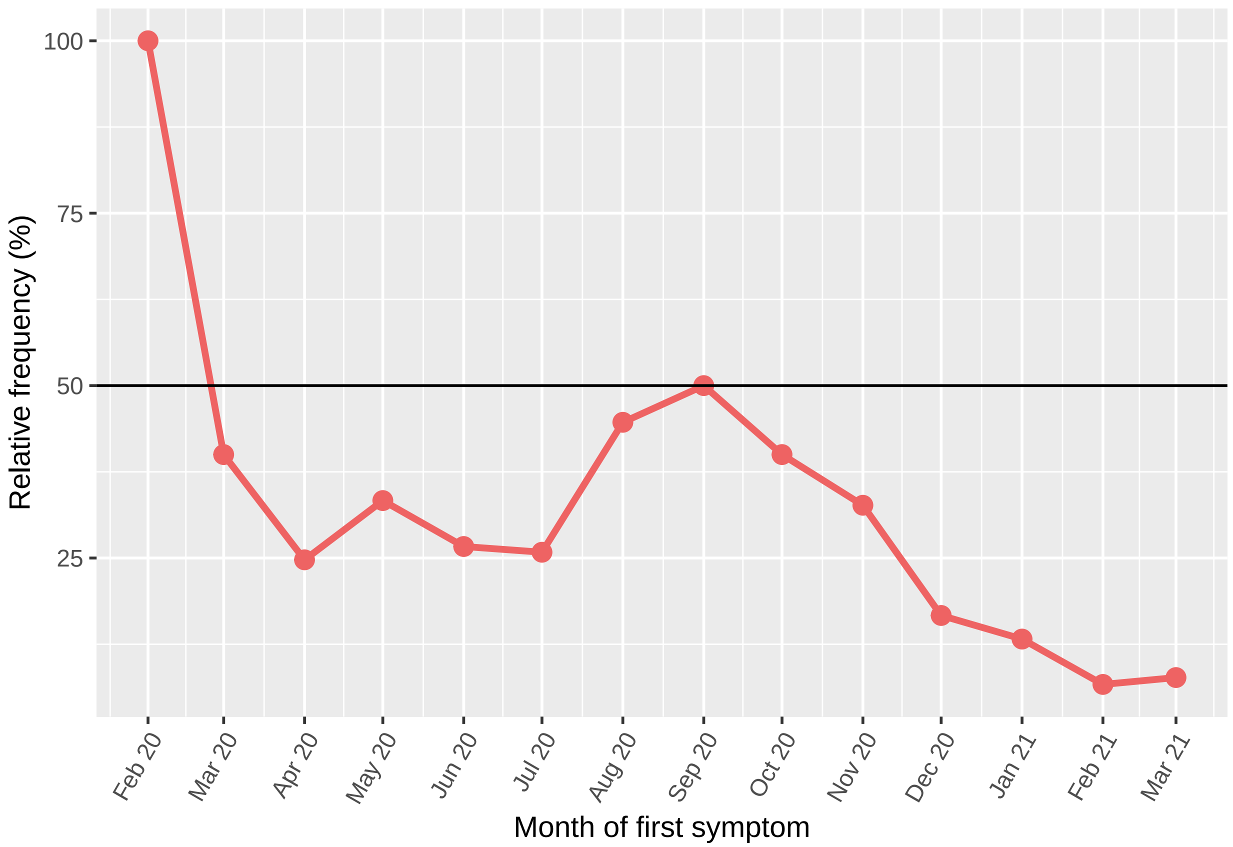


```
ggsave("epidemiologic_month",dpi="print",device="tiff")


#Values
d11 <- table(d1$month_year,d1$group)
d11 <- as.data.frame(d11)
d11 <- data.frame(str_split(d11$Var1,"/",simplify=TRUE),d11,G4$Freq*100)
d11 <- d11 %>% arrange(X2,X1)
d11$Var1 <- NULL
d11 <- rename(d11,"Month"="X1","Year"="X2","group"="Var2","n"="Freq","%"="G4.Freq...100")
d11
```

```
##    Month Year       group  n          %
## 1     02 2020 unspecified  3 100.000000
## 2     02 2020    covid-19  0   0.000000
## 3     03 2020 unspecified 10  40.000000
## 4     03 2020    covid-19 15  60.000000
## 5     04 2020 unspecified 24  24.742268
## 6     04 2020    covid-19 73  75.257732
## 7     05 2020 unspecified 43  33.333333
## 8     05 2020    covid-19 86  66.666667
## 9     06 2020 unspecified 28  26.666667
## 10    06 2020    covid-19 77  73.333333
## 11    07 2020 unspecified 23  25.842697
## 12    07 2020    covid-19 66  74.157303
## 13    08 2020 unspecified 21  44.680851
```

```
## 14    08 2020    covid-19  26  55.319149
## 15    09 2020 unspecified  18  50.000000
## 16    09 2020    covid-19  18  50.000000
## 17    10 2020 unspecified  10  40.000000
## 18    10 2020    covid-19  15  60.000000
## 19    11 2020 unspecified  16  32.653061
## 20    11 2020    covid-19  33  67.346939
## 21    12 2020 unspecified   9  16.666667
## 22    12 2020    covid-19  45  83.333333
## 23    01 2021 unspecified  11  13.253012
## 24    01 2021    covid-19  72  86.746988
## 25    02 2021 unspecified  10   6.666667
## 26    02 2021    covid-19 140  93.333333
## 27    03 2021 unspecified  24   7.667732
## 28    03 2021    covid-19 289  92.332268
```

```
write_csv(d11, "dados_figura2.csv")
```
